# Guideline appraisal with AGREE II: Systematic review of the current evidence on how users handle the 2 overall assessments

**DOI:** 10.1371/journal.pone.0174831

**Published:** 2017-03-30

**Authors:** Wiebke Hoffmann-Eßer, Ulrich Siering, Edmund A. M. Neugebauer, Anne Catharina Brockhaus, Ulrike Lampert, Michaela Eikermann

**Affiliations:** 1 Institute for Quality and Efficiency in Health Care (IQWiG), Cologne, Germany; 2 Institute for Research in Operative Medicine (IFOM), University of Witten/Herdecke, Campus Cologne, Cologne, Germany; 3 Brandenburg Medical School – Theodor Fontane Neuruppin, Germany & University of Witten/Herdecke, Witten/Herdecke, Germany; 4 Medical Advisory Service of the German Social Health Insurance (MDS), Essen, Germany; National Natural Science Foundation of China, CHINA

## Abstract

**Introduction:**

The Appraisal of Guidelines for Research & Evaluation (AGREE) II instrument is the most commonly used guideline appraisal tool. It includes 23 appraisal criteria (items) organized within 6 domains and 2 overall assessments (1. overall guideline quality; 2. recommendation for use). The aim of this systematic review was twofold. Firstly, to investigate how often AGREE II users conduct the 2 overall assessments. Secondly, to investigate the influence of the 6 domain scores on each of the 2 overall assessments.

**Materials and methods:**

A systematic bibliographic search was conducted for publications reporting guideline appraisals with AGREE II. The impact of the 6 domain scores on the overall assessment of guideline quality was examined using a multiple linear regression model. Their impact on the recommendation for use (possible answers: “yes”, “yes, with modifications”, “no”) was examined using a multinomial regression model.

**Results:**

118 relevant publications including 1453 guidelines were identified. 77.1% of the publications reported results for at least one overall assessment, but only 32.2% reported results for both overall assessments. The results of the regression analyses showed a statistically significant influence of all domains on overall guideline quality, with Domain 3 (rigour of development) having the strongest influence. For the recommendation for use, the results showed a significant influence of Domains 3 to 5 (“yes” vs. “no”) and Domains 3 and 5 (“yes, with modifications” vs. “no”).

**Conclusions:**

The 2 overall assessments of AGREE II are underreported by guideline assessors. Domains 3 and 5 have the strongest influence on the results of the 2 overall assessments, while the other domains have a varying influence. Within a normative approach, our findings could be used as guidance for weighting individual domains in AGREE II to make the overall assessments more objective. Alternatively, a stronger content analysis of the individual domains could clarify their importance in terms of guideline quality. Moreover, AGREE II should require users to transparently present how they conducted the assessments.

## Introduction

According to the definition of the US Institute of Medicine, “clinical practice guidelines are statements that include recommendations intended to optimize patient care that are informed by a systematic review of evidence and an assessment of the benefits and harms of alternative care options.” [1].

Various studies have shown that guidelines can improve health care [2–14]. However, their quality is variable and therefore their recommendations are often inconsistent [15–24].

In order to be able to use guidelines as a reliable basis for decision-making, their quality, i.e. their methodological rigour and transparency, needs to be ensured. Guideline appraisal tools are applied for this purpose. Forty such tools covering varying dimensions of guideline quality were identified in a systematic review published in 2013 [25], of which 6 contain a quantitative assessment of overall guideline quality.

In 2003, an international group of guideline developers and researchers developed the Appraisal of Guidelines for Research & Evaluation (AGREE) instrument [15]. The revised version, AGREE II [26], was published in 2009 and is currently the most commonly applied and comprehensively validated guideline appraisal tool worldwide [17–19]. It consists of 23 appraisal criteria (items) organized into 6 domains (Table 1), each of which “captures a unique dimension of guideline quality” [16]. The items within each domain are rated on a 7-point scale (“strongly disagree” to “strongly agree”).

**Table 1:** Items and domains of the AGREE II instrument^a^.

Item	Content	Domain
1	The overall objective(s) of the guideline is (are) specifically described.	Scope and Purpose
2	The health question(s) covered by the guideline is (are) specifically described.	
3	The population (patients, public, etc.) to whom the guideline is meant to apply is specifically described.	
4	The guideline development group includes individuals from all relevant professional groups.	Stakeholder Involvement
5	The views and preferences of the target population (patients, public, etc.) have been sought.	
6	The target users of the guideline are clearly defined.	
7	Systematic methods were used to search for evidence.	Rigour of Development
8	The criteria for selecting the evidence are clearly described.	
9	The strengths and limitations of the body of evidence are clearly described.	
10	The methods for formulating the recommendations are clearly described.	
11	The health benefits, side effects, and risks have been considered in formulating the recommendations.	
12	There is an explicit link between the recommendations and the supporting evidence.	
13	The guideline has been externally reviewed by experts prior to its publication.	
14	A procedure for updating the guideline is provided.	
15	The recommendations are specific and unambiguous.	Clarity of Presentation
16	The different options for management of the condition or health issue are clearly presented.	
17	Key recommendations are easily identifiable.	
18	The guideline describes facilitators and barriers to its application.	Applicability
19	The guideline provides advice and/or tools on how the recommendations can be put into practice.	
20	The potential resource implications of applying the recommendations have been considered.	
21	The guideline presents monitoring and/or auditing criteria.	
22	The views of the funding body have not influenced the content of the guideline.	Editorial Independence
23	Competing interests of guideline development group members have been recorded and addressed.	

^a^: Extracted from the AGREE II instrument

In addition, AGREE II includes 2 global rating items (overall assessments). In the first overall assessment, the overall guideline quality is rated on a 7-point scale (“lowest possible quality” to “highest possible quality”). In the second overall assessment, a recommendation is provided on whether to use the guideline in practice or not (recommendation for use: “yes”, “yes with modifications”, “no”). Both assessments should consider the 23 items evaluated beforehand and the resulting domain scores, but should not be calculated from them.

It has not yet been investigated in the literature how often AGREE II users conduct the 2 overall assessments. For this reason, it is unclear whether these assessments actually represent separate assessments (as specified by AGREE II) or whether users simply calculate the overall scores directly from the domain scores.

On the basis of recent publications on guideline appraisals, the aim of this systematic review was twofold. Firstly, to investigate how AGREE II users handle the 2 overall assessments, that is, how often they conduct them. Secondly, to investigate the influence of the 6 domain scores on each of the 2 overall assessments (1. overall guideline quality; 2. recommendation for use).

## Materials and methods

A systematic search for relevant (primary and secondary) publications was conducted in MEDLINE, EMBASE, the Database of Abstracts of Reviews of Effects (Other Reviews), and the Health Technology Assessment Database (Technology Assessments).

Amongst others, the following search terms were used: “practice guidelines as topic”, “AGREE instrument” and “methodological guideline appraisal”. The full list of search terms is included in the search strategy (see supporting information S1 File), which was developed by an information specialist. The search was conducted in January 2016.

German- and English-language publications reporting results of at least one guideline appraisal with AGREE II were considered. These results had to include all 6 standardized domain scores of each guideline appraised.

**Fig 1:**
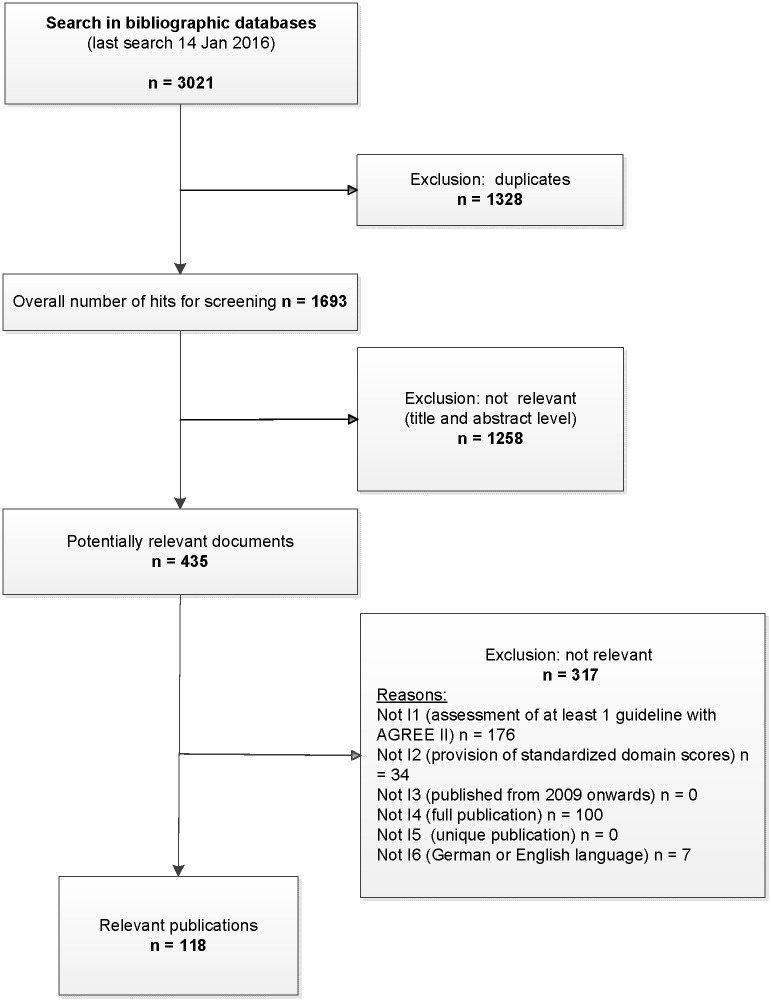
Results of the systematic literature search.

The screening of titles and abstracts and subsequently of full texts was performed by 2 authors independently of one another. 96 discrepancies in the screening of title and abstracts and 128 discrepancies in the screening of full texts were resolved by discussion between both authors (see Fig 1).

### Data extraction and analysis

The results of the AGREE II appraisals (standardized domain scores, and, if available, results of the overall assessments) were extracted from the publications included. In addition, the main characteristics of these publications were extracted, namely, the aim of the publication, the number of assessors, the number of guidelines appraised with AGREE II, the publication dates of the guidelines included in the relevant publications, as well as the guideline topics (S4 File).

Further information was also extracted from the publications (S5 File). This referred to whether overall assessment 1 (overall guideline quality) and/or overall assessment 2 (recommendations for use) had been conducted or not. If yes, it was also examined whether the requirements of AGREE II had been followed.

Data extraction and analysis were performed by one reviewer and checked by another. Any discrepancies were resolved by discussion between them. It was then checked how often the overall assessments had been performed in the guideline appraisals.

The impact of the 6 standardized domain scores (independent variables) on the overall assessment of guideline quality (dependent variable) was examined using a multiple linear regression model. Guideline appraisals were excluded from the multiple linear regression analysis if a standardized domain score was not available for all 6 domains. Similarly, guidelines were excluded whose overall guideline quality had been calculated from the standardized domain scores using the mean values, as this approach is not recommended by AGREE II. The inclusion of such guideline appraisals could have biased our results concerning the influence of the 6 domains on the 2 overall assessments, as this influence would have been determined by calculation, not by evaluation.

In a second analysis, the impact of the 6 standardized domain scores (independent variables) on the recommendation for use (dependent variable) was examined using a multinomial regression model. Guideline appraisals were excluded from the multinomial regression if they did not contain data on standardized domain scores for all 6 domains.

It is possible to receive inconsistent information on the recommendations for use due to independent evaluations by several assessors (e.g. both “yes, with modifications” and “no” or both “yes” and “yes, with modifications”). In these cases, the recommendation for use was allocated to the category “yes, with modifications”. In addition, guideline appraisals were excluded from the analysis if no allocation of the recommendation for use to one of the 3 categories (“yes”, “yes, with modifications”, “no”) was meaningful. This could be the case if inconsistent recommendations for use were provided for the same guideline, such as both “yes” and “no”, or all 3 categories (“yes”, “yes, with modifications”, “no”).

Due to the multiple comparisons performed, we also present adjusted p-values for each regression analysis according to Benjamini and Hochberg [27] to control for the false discovery rate and maintain an overall significance level of 5%. The decision on whether a domain had a significant influence on the overall assessments or not was based on this adjusted p-value. The data were analysed with SPSS Statistics 18 and SAS 9.3.

## Results

### Selection of relevant publications

The systematic search in bibliographic databases identified a total of 3021 publications, of which 435 were screened in full text; 118 fulfilled the inclusion criteria (Fig 1). The supporting information contains the list of publications included (S2 File) and excluded (S3 File), with the reasons for exclusion, as well as the main characteristics of the guidelines appraised in the publications (S4 File).

### Results for the first research question

#### Conduct of overall assessments

**Fig 2:**
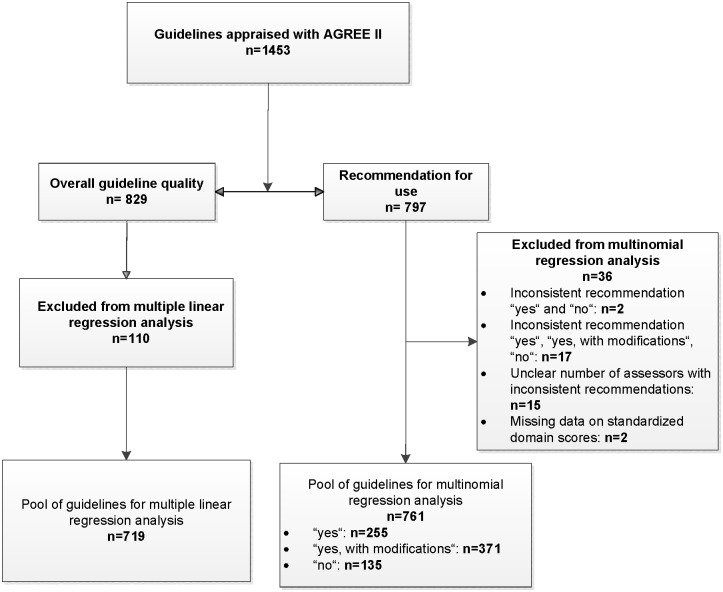
Guideline pool for the multiple linear and multinomial regression analyses.

91 (77.1%) of the 118 eligible publications reported results for at least one overall assessment of which 38 (32.2%) reported both overall assessments, 32 (27.1%) reported only overall assessment 1 (overall guideline quality), and 21 (17.8%) reported only overall assessment 2 (recommendation for use); see S5 File. The 91 publications included 1453 guidelines appraised with AGREE II (Fig 2).

70 publications (38 + 32) therefore included at least one result on the assessment of overall guideline quality, while 59 publications (38 + 21) included at least one result on the assessment of the recommendation for use.

#### Overall assessment 1 (overall guideline quality)

The overall guideline quality had been assessed for 829 (57.1%) of the 1453 guidelines.

In 10 (14.3%) of the 70 publications reporting overall guideline quality, the authors apparently calculated the overall score from the mean scores of the 6 standardized domain scores [28–37]; see S5 File. The data from these 10 publications, which contained 110 guidelines, were not considered in the multiple regression analysis.

719 (49.5%) guidelines thus formed the total pool for the analysis of the association between standardized domain scores and overall guideline quality (Fig 2).

#### Overall assessment 2 (recommendation for use)

A recommendation for use was provided by the assessors for 797 (54.9%) of the 1453 guidelines. All guideline appraisals (n = 797) were performed by between 2 and 11 assessors independently of one another; different recommendations for use were therefore provided for the same guideline (e.g. both “yes, with modifications” and “no” or both “yes” and “yes, with modifications”). In such cases (n = 53), the assessment was allocated to the category “yes, with modifications”.

In addition, further inconsistent information on the recommendations for use was provided for the same guideline by the different assessors: both “yes” and “no” (n = 2) as well as all 3 categories (“yes”, “yes, with modifications”, “no”; n = 17). Moreover, in one publication the number of assessors was not clear for the guidelines with inconsistent recommendations (n = 15); these results could not be allocated to any of the 3 categories above and were thus not included in the multinomial regression analysis. Likewise, 2 guideline appraisals were excluded, since they did not contain data on standardized domain scores for all 6 domains. A total of 36 (4.5%) guidelines were thus excluded from the multinomial regression analysis (Fig 2).

Overall, consistent recommendations for use were provided for 708 (88.8%) of the 797 guidelines with a recommendation for use. Ultimately, 761 (52.4%) guidelines formed the pool for the multinomial regression analysis: 255 (33.5%), 371 (48.8%), and 135 (17.7%) were allocated to the categories “yes”, “yes, with modifications”, and “no” respectively (Fig 2).

### Results for the second research question

#### Evaluation of model assumptions and correlations between independent variables: Multiple regression analysis

No major violations were shown in the evaluation of the model assumptions of the multiple linear regression analysis (S6 File). Weak (r < 0.5) to moderate (0.5 ≤ r < 0.8) correlations were shown for all pairs of independent variables considered (domains 1 to 6).

#### Evaluation of model assumptions and correlations between independent variables: Multinomial regression analysis

Weak (r < 0.5) to moderate (0.5 ≤ r < 0.8) correlations were shown for all pairs of independent variables considered in the multinomial regression analysis; S7 File.

#### Influence of the 6 domains on the results of overall assessment 1 (overall guideline quality)

All domains had a statistically significant influence (adjusted p-value < 0.05) on overall guideline quality (Table 2). Domain 3 had the strongest influence (ß = 0.300; adjusted p-value < 0.001), followed by Domain 4 (ß = 0.203; adjusted p-value < 0.001) and Domain 1 (ß = 0.175; adjusted p-value < 0.001), as well as Domain 5 (ß = 0.163; adjusted p-value < 0.001), Domain 6 (ß = 0.065; adjusted p-value < 0.001), and Domain 2 (ß = 0.062; adjusted p-value = 0.018).

**Table 2:** Results of the multiple regression analysis (independent variable: Overall guideline quality).

Predictors	Unstandardized coefficients		95% confidence interval for B		t	P-value	Adjusted P-value (sig. < 0.05)
	B	Standard error	Lower bound	Upper bound			
Intercept	5.591	1.753			3.19	0.001	
Domain 1 (scope and purpose)	.175	0.026	.125	.226	6.784	< 0.001	< 0.001
Domain 2 (stakeholder involvement)	.062	0.026	.011	.114	2.381	0.018	0.018
Domain 3 (rigour of development)	.300	0.025	.250	.350	11.796	< 0.001	< 0.001
Domain 4 (clarity of presentation)	.203	0.027	.150	.255	7.583	< 0.001	< 0.001
Domain 5 (applicability)	.163	0.021	.123	.204	7.913	< 0.001	< 0.001
Domain 6 (editorial independence)	.065	0.017	.032	.099	3.841	< 0.001	< 0.001

Dependent variable: overall guideline quality; adjusted R2: 0.732

#### Influence of the 6 domains on the results of overall assessment 2 (recommendation for use)

According to AGREE II, there are 3 categories for the recommendation for use ("yes", "yes, with modifications", "no"). For the multinomial regression analysis, the category “no” of the recommendation for use was chosen as the reference category, resulting in 2 comparisons: “yes” vs. “no” and “yes, with modifications” vs. “no”.

The comparison of the categories “yes” and “no” showed that Domains 3 to 5 had a significant influence (adjusted p-value < 0.05) on whether the use of a guideline was recommended (Table 3). Domain 3 had the strongest influence (ß = 0.109; adjusted p-value < 0.001) followed by Domains 4 (ß = 0.046; adjusted p-value < 0.001), and 5 (ß = 0.022; adjusted p-value = 0.028). With an increase in the standardized domain score of Domain 3 by 10 percentage points, there was almost a 3-fold increase in the ratio of guidelines recommended for use versus those not recommended (if all other variables remained unchanged). With the same increase in Domains 4 and 5, there was almost a 1.6 and 1.3-fold increase in this ratio, respectively.

**Table 3:** Results of the multinomial regression analysis (independent variable: Recommendation for use for the categories “yes” vs. “no”).

Parameter	Estimate	Standard error	Wald chi-square	P-value	Adjusted p-value (sig. < 0.05)	OR^a^	95% confidence interval for OR^a^	
							Lower bound	Upper bound
Intercept (recommended)	-9.744	0.856	129.729	< 0.001				
Domain 1 (scope and purpose)	0.013	0.009	2.059	0.151	0.227	1.140	0.954	1.367
Domain 2 (stakeholder involvement)	0.013	0.010	1.603	0.206	0.247	1.135	0.933	1.381
Domain 3 (rigour of development)	0.109	0.011	93.824	< 0.001	< 0.001	2.963	2.395	3.719
Domain 4 (clarity of presentation)	0.046	0.010	20.521	< 0.001	< 0.001	1.581	1.301	1.934
Domain 5 (applicability)	0.022	0.009	6.026	0.014	0.028	1.250	1.048	1.498
Domain 6 (editorial independence)	0.003	0.006	0.200	0.657	0.657	1.029	0.909	1.166

^a^: The OR corresponds to the change in the respective domain score by 10 percentage points.

Dependent variable: recommendation for use; Reference category: “no”.

The comparison of the categories “yes, with modifications” and “no” showed that Domains 3 and 5 had a significant influence on whether the use of a guideline was recommended with modifications (Table 4). Domain 3 had the strongest influence (ß = 0.061; adjusted p-value < 0.001), followed by Domain 5 (ß = 0.022; adjusted p-value = 0.019). With an increase in the standardized domain score of Domain 3 by 10 percentage points, there was about a 1.8-fold increase in the ratio of guidelines recommended for use with modifications versus those not recommended (if all other variables remained unchanged). With the same increase in Domain 5, there was a 1.2-fold increase in this ratio.

**Table 4:** Results of the multinomial regression analysis (independent variable: Recommendation for use for the categories; “yes, with modifications” vs. “no”).

Parameter	Estimate	Standard error	Wald chi-square	P-value	Adjusted p-value (sig. < 0.05)	OR^a^	95% confidence interval for OR^a^	
							Lower bound	Upper bound
Intercept (recommended, with modifications)	-3.224	0.472	46.584	< 0.001				
Domain 1 (scope and purpose)	0.014	0.006	4.765	0.029	0.058	1.146	1.014	1.297
Domain 2 (stakeholder involvement)	0.005	0.008	0.303	0.582	0.699	1.047	0.889	1.233
Domain 3 (rigour of development)	0.061	0.009	43.945	< 0.001	< 0.001	1.843	1.549	2.226
Domain 4 (clarity of presentation)	0.012	0.007	3.438	0.064	0.096	1.132	0.994	1.293
Domain 5 (applicability)	0.022	0.008	7.497	0.006	0.019	1.246	1.068	1.465
Domain 6 (editorial independence)	0.000	0.005	0.009	0.926	0.926	1.005	0.908	1.114

^a^: The OR corresponds to the change in the respective domain score by 10 percentage points.

Dependent variable: recommendation for use; Reference category: “no”.

## Discussion

### Main findings

The aim of this systematic review was twofold. Firstly, to investigate how AGREE II users handle the 2 overall assessments (1. overall guideline quality, 2. recommendation for use), that is, how often they conduct them. Secondly, to investigate the influence of the 6 domain scores on each of the 2 overall assessments.

Even though the assessment of overall guideline quality and the recommendation for use are standard components of AGREE II, they are underreported: 77.1% of the eligible publications reported results for at least one overall assessment, but only 32.2% reported results for both overall assessments.

Regarding the influence of domains, both regression analyses showed that Domain 3 (rigour of development) had the strongest influence on the 2 overall assessments. Furthermore, all analyses showed a statistically significant influence of Domain 5 (applicability) on both overall assessments. For Domain 4 (clarity of presentation), the results were statistically significant for the multiple linear regression analysis (overall guideline quality), as well as for part of the multinomial regression analysis (recommendation for use: “yes” vs. “no”); in both of these analyses this domain showed the second strongest influence.

### Relation to other studies

The strong influence of Domain 3 on the 2 overall assessments is not surprising, as previous research suggests that this domain is a stronger indicator of guideline quality than the other domains [16, 38], a high score indicating minimum bias and evidence-based guideline development [38]. On the other hand, a low score indicates serious methodological problems, for instance, a lack of methodological expertise in guideline developing teams or an inadequate systematic search due to a lack of resources [16].

The results for Domain 5 can be explained by what Gagliardi et al. note in their systematic review of guideline applicability. The items of Domain 5 refer to facilitators and barriers of guideline implementation, monitoring or audit criteria, and implementation instructions / tools. According to Gagliardi et al., for the latter there is evidence of association with guideline use: If a guideline is insufficiently implemented in clinical practice because of inadequate implementation instructions, this “contributes to omission of beneficial therapies, preventable harm, suboptimal patient outcomes or experiences, or waste of resources” [39].

However, the items in Domain 5 need to be distinguished from the specific applicability of a guideline in clinical practice (e.g. in the care of a certain patient population); a guideline may not be applicable to a specific context, but may still receive a high score in Domain 5 [40].

The strong influence of Domain 4 is not surprising either, as “[t]he main advantage of a well-reported guideline is that flaws in the methodology are more easily detected, so that inherent biases can be considered more explicitly and scrutinized by the potential users” [41].

### Calculation of overall guideline quality

In contrast to the requirements of AGREE II, in 10 publications the overall assessment 1 (overall guideline quality) was calculated as the mean of the 6 standardized domain scores [28–37]; it is thus highly likely that no separate assessment of overall guideline quality independent of the domain scores was performed. According to the AGREE II requirements the assessment of the overall quality of a guideline should be subjective. This is not possible if the assessment is based on a calculation, as each of the 6 domains would have a similar influence on the overall quality. Such an approach would thus not have answered our second research question. As previously stated, this approach is not recommended by AGREE II and these 10 publications were excluded from further analysis.

### Strengths and limitations

#### Language restrictions

The search for relevant publications was limited to German and English-language publications. Since AGREE II is an internationally recognized and validated instrument that has been translated into several languages not considered here, potentially relevant publications in other languages were not taken into account in our analysis, which may have led to language bias.

#### Choice of regression model

The data on the overall assessment 2 (recommendation for use) were ordinally scaled. Initially we attempted to adjust an ordinally scaled regression model to answer our objective. However, an evaluation of the model assumptions showed that the assumption of proportional odds was not fulfilled; this model was thus inappropriate and could have led to misleading results [42].

#### Independent variables

The adjusted determination coefficient (R2) for the multiple linear regression analysis shows that 73.2% of the variance in the overall guideline quality can be explained by the independent variables (standardized domain scores of the 6 domains) considered in the analysis (Table 2 and S6 File). The determination coefficient (R2) in the multinomial regression analysis was 58.2% (S7 File).

Besides the standardized domain scores, no further independent variables were considered in the 2 regression models. Further research would need to investigate to what extent further guideline characteristics affect the results of the regression models (e.g. guideline topic, country of origin, publisher, publication date, profession and level of experience of AGREE II users, and whether or not a consensus procedure was conducted in the event of deviating appraisals). In addition, factors affecting the internal validity of a guideline (e.g. consistency of recommendations) that go beyond the methodological aspects of an AGREE appraisal could play a role.

#### Sample size for regression analyses

According to Schneider et al. 2010, at least 20 observations should be available for each independent variable [43]. The multiple linear regression model included 6 independent variables (domains 1 to 6); therefore the minimum sample size of 6x20 = 120 observations (guidelines) had to be available in the multiple linear regression analysis. This analysis was based on 719 guidelines appraised with AGREE II. An insufficient sample size could falsely create strong associations between variables. However, the above estimate applies only to the multiple linear regression analysis; it is not directly applicable to the multinomial regression analysis.

#### Correlation of the independent variables in the regression analyses

The correlation of the independent variables should be considered in the overall interpretation of the results of the regression analyses. A statistically non-significant result for an independent variable (standardized domain score) does not necessarily mean a lack of association with the dependent variable (overall guideline quality or recommendation for use). If 2 independent variables correlate with each other, this can lead to a situation where one of these variables does not contribute additional information to the regression analysis [43].

#### Strengths and limitations of AGREE II

Due to the wide range of domain items, the AGREE II instrument offers the opportunity to systematically, specifically and objectively evaluate the quality of guidelines from all specialties [44]. However, as stated above and also noted by several other researchers [44–49] AGREE II lacks detailed information on how to perform the 2 overall assessments. In addition, several researchers emphasize that these assessments are subjective [31, 44, 48, 50, 51]; some regard it as a weakness of AGREE II that items or domains are not weighted, but are all considered equally [45–47, 50, 52]. Before appraising a guideline with AGREE II, Lytras et al. thus propose to weight domain items [47].

The results of an AGREE II appraisal should be viewed with caution, as different guideline assessors may interpret the items and scoring system differently [53].

Due to the problems described above, some researchers criticize that AGREE II allows no clear distinction between high- and low-quality guidelines [33, 47, 48, 54]. Several researchers use cut-offs to distinguish between high and low quality [32, 44, 50, 55–60]. This shows that AGREE II users would welcome such a clear distinction, but the instrument currently does not fulfil this requirement.

### Implications for further research

Besides the standardized domain scores, no further independent variables were considered in the 2 regression models. Future research would need to investigate to what extent additional guideline characteristics potentially affect the results of the regression models (e.g. guideline topic, country of origin, publisher, publication date, profession and level of experience of AGREE II users, and whether or not a consensus procedure was conducted in the event of deviating appraisals). In addition, factors affecting the internal validity of a guideline (e.g. consistency of recommendations) that go beyond the methodological aspects of an AGREE appraisal might play a role.

## Conclusion

The 2 overall assessments of the AGREE II instrument are underreported by guideline assessors. Domains 3 and 5 have the strongest influence on the results of the 2 overall assessments, while the other domains have a varying influence.

As a normative approach, the results of our study could be used as guidance for weighting individual domains in AGREE II, an approach already proposed by authors of guideline appraisals. Consequently, the 2 overall assessments would be performed in a more objective manner. Alternatively, a stronger content analysis of the individual domains or their items could be carried out to clarify their importance in terms of the quality of a guideline.

In addition, AGREE II should require users to transparently present how they performed the 2 overall assessments. This particularly refers to the recommendation for use; guideline assessors should explain on which criteria their recommendation is based, allowing readers to form their own judgement on whether they would have provided the same recommendation in the same healthcare setting.

## Supporting information

S1 File Search strategy. (PDF)

S2 File Publications included. (PDF)

S3 File Publications excluded (organized by reasons for exclusion). (PDF)

S4 File Characteristics of guidelines included in the publications. (PDF)

S5 File Information on the conduct of the overall assessments according to AGREE II. (PDF)

S6 File Statistics for the multiple regression analysis. (PDF)

S7 File Assessment of model quality of the multinomial regression analysis. (PDF)

S8 File PRISMA-checklist. (PDF)
