# Supplementary material for: Guideline appraisal with AGREE II: Systematic review of the current evidence on how users handle the 2 overall assessments
Source: PLoS One. 2017 Mar 30;12(3):e0174831. doi: 10.1371/journal.pone.0174831 (PMC5373625; doi:10.1371/journal.pone.0174831)
Supplement: S1 File — (PDF) [file pone.0174831.s001.pdf]

S1: Search strategy

1. EMBASE

Search interface: Ovid

Embase 1974 to 2016 January 13

#	**Searches**
1	exp practice guideline/
2	guideline*.ti,ab.
3	or/1-2
4	(agree* adj5 (instrument* or criteria*)).ti,ab.
5	(apprais* adj7 evaluat*).ti,ab.
6	(quality* adj5 (tool* or instrument*)).ti,ab.
7	or/4-6
8	3 and 7
9	limit 8 to ((english or german) and yr="2009 -Current")
10	9 not medline*.cr.

2. MEDLINE

Search interface: Ovid

- Ovid MEDLINE(R) In-Process & Other Non-Indexed Citations January 13, 2016
- Ovid MEDLINE(R) 1946 to January Week 1 2016
- Ovid MEDLINE(R) Daily Update January 13, 2016

#	**Searches**
1	Practice Guidelines as Topic/

2	guideline*.ti,ab.
3	or/1-2
4	(agree* adj5 (instrument* or criteria*)).ti,ab.
5	(apprais* adj7 evaluat*).ti,ab.
6	(quality* adj5 (tool* or instrument*)).ti,ab.
7	or/4-6
8	3 and 7
9	limit 8 to ((english or german) and yr="2009 -Current")

3. PubMed

Search interface: NLM

- PubMed - as supplied by publisher
- PubMed - in process
- PubMed – OLDMEDLINE
- PubMed – pubmednotmedline

**Search**	**Query**
#1	Search guideline*[tiab]
#2	Search ((agree*[tiab] AND (instrument*[tiab] OR criteria*[tiab])))
#3	Search (apprais*[tiab] AND evaluat*[tiab])
#4	Search ((quality*[tiab] AND (tool*[tiab] OR instrument*[tiab])))
#5	Search (#2 OR #3 OR #4)
#6	Search (#1 AND #5)
#7	Search (#6 not medline[sb])
#8	Search (#6 not medline[sb]) Filters: Publication date from 2009/01/01 to 2016/12/31; English; German

4. The Cochrane Library

Search interface: Wiley

- Database of Abstracts of Reviews of Effect : Issue 2 of 4, April 2015
- Health Technology Assessment Database : Issue 4 of 4, October 2015

ID	Search
#1	MeSH descriptor: [Practice Guidelines as Topic] explode all trees
#2	guideline*
#3	#1 or #2
#4	(agree* near/5 (instrument* or criteria*))
#5	(apprais* near/7 evaluat*)
#6	(quality* near/5 (tool* or instrument*))
#7	#4 or #5 or #6
#8	#3 and #7 Publication Year from 2009 to 2016, in Other Reviews and Technology Assessments
