# Supplementary material for: Guideline appraisal with AGREE II: Systematic review of the current evidence on how users handle the 2 overall assessments
Source: PLoS One. 2017 Mar 30;12(3):e0174831. doi: 10.1371/journal.pone.0174831 (PMC5373625; doi:10.1371/journal.pone.0174831)
Supplement: S2 File — (PDF) [file pone.0174831.s002.pdf]

S2: Publications included

[1] Abdelsattar ZM, Reames BN, Regenbogen SE, Hendren S, Wong SL. Critical evaluation of the scientific content in clinical practice guidelines. Cancer. 2015;121:783-9.

[2] Acuna-Izcaray A, Sanchez-Angarita E, Plaza V, Rodrigo G, Montes de Oca M, Gich I, et al. Quality assessment of asthma clinical practice guidelines: a systematic appraisal. Chest. 2013;144:390-7.

[3] Agrawal S, Jones B, Barnes R, Kibbler C, Millen M, Ashcroft M, et al. A practical critique of antifungal treatment guidelines for haemato-oncologists. Crit Rev Microbiol. 2012;38:203-16.

[4] Al-Ansary LA, Tricco AC, Adi Y, Bawazeer G, Perrier L, Al-Ghonaim M, et al. A systematic review of recent clinical practice guidelines on the diagnosis, assessment and management of hypertension. PLoS One. 2013;8:e53744.

[5] Altman RD, Schemitsch E, Bedi A. Assessment of clinical practice guideline methodology for the treatment of knee osteoarthritis with intra-articular hyaluronic acid. Semin Arthritis Rheum. 2015;45:132-9.

[6] Alvarez-Vargas ML, Galvez-Olortegui JK, Galvez-Olortegui TV, Sosa-Rosado JM, Camacho-Saavedra LA. Clinical practice guidelines in hypertension: a review. Medwave. 2015;15:e6290.

[7] Arevalo-Rodriguez I, Pedraza OL, Rodriguez A, Sanchez E, Gich I, Sola I, et al. Alzheimer's disease dementia guidelines for diagnostic testing: a systematic review. Am J Alzheimers Dis Other Demen. 2013;28:111-9.

[8] Armstrong JJ, Rodrigues IB, Wasiuta T, MacDermid JC. Quality assessment of osteoporosis clinical practice guidelines for physical activity and safe movement: an AGREE II appraisal. Arch Osteoporos. 2016;11:6.

[9] Avin KG, Hanke TA, Kirk-Sanchez N, McDonough CM, Shubert TE, Hardage J, et al. Management of falls in community-dwelling older adults: clinical guidance statement from the Academy of Geriatric Physical Therapy of the American Physical Therapy Association. Phys Ther. 2015;95:815-34.

[10] Barber CE, Smith A, Esdaile JM, Barnabe C, Martin LO, Faris P, et al. Best practices for cardiovascular disease prevention in rheumatoid arthritis: a systematic review of guideline recommendations and quality indicators. Arthritis Care Res (Hoboken). 2015;67:169-79.

[11] Bekkering GE, Aertgeerts B, Asueta-Lorente JF, Autrique M, Goossens M, Smets K, et al. Practitioner review: evidence-based practice guidelines on alcohol and drug misuse among adolescents; a systematic review. J Child Psychol Psychiatry. 2014;55:3-21.

[12] Binepal N, Lemyre B, Dunn S, Daboval T, Aglipay M, Leduc S, et al. Systematic review and quality appraisal of international guidelines on perinatal care of extremely premature infants. Curr Pediatr Rev. 2015;11:126-34.

[13] Birken SA, Ellis SD, Walker JS, DiMartino LD, Check DK, Gerstel AA, et al. Guidelines for the use of survivorship care plans: a systematic quality appraisal using the AGREE II instrument. Implement Sci. 2015;10:63.

[14] Bragge P, Pattuwage L, Marshall S, Pitt V, Piccenna L, Stergiou-Kita M, et al. Quality of guidelines for cognitive rehabilitation following traumatic brain injury. J Head Trauma Rehabil. 2014;29:277-89.

[15] Brosseau L, Rahman P, Poitras S, Toupin-April K, Paterson G, Smith C, et al. A systematic critical appraisal of non-pharmacological management of rheumatoid arthritis with Appraisal of Guidelines for Research and Evaluation II. PLoS One. 2014;9:e95369.

[16] Brosseau L, Rahman P, Toupin-April K, Poitras S, King J, De Angelis G, et al. A systematic critical appraisal for non-pharmacological management of osteoarthritis using the Appraisal of Guidelines Research and Evaluation II instrument. PLoS One. 2014;9:e82986.

[17] Burda BU, Chambers AR, Johnson JC. Appraisal of guidelines developed by the World Health Organization. Public Health. 2014;128:444-74.

[18] Burnett HF, Tanoshima R, Chandranipapongse W, Madadi P, Ito S, Ungar WJ. Testing for thiopurine methyltransferase status for safe and effective thiopurine administration: a systematic review of clinical guidance documents. Pharmacogenomics J. 2014;14:493-502.

[19] Cassis L, Cortes-Saladelafont E, Molero-Luis M, Yubero D, Gonzalez MJ, Herrero AO, et al. Review and evaluation of the methodological quality of the existing guidelines and recommendations for inherited neurometabolic disorders. Orphanet J Rare Dis. 2015;10:164.

[20] Castellani A, Girlanda F, Barbui C. Rigour of development of clinical practice guidelines for the pharmacological treatment of bipolar disorder: systematic review. J Affect Disord. 2015;2015:45-50.

[21] Chen Y, Hu S, Wu L, Fang X, Xu W, Shen G. Clinical practice guidelines for hypertension in China: a systematic review of the methodological quality. BMJ Open. 2015;5:e008099.

[22] Chua ME, Mendoza J, See M, Esmena E, Aguila D, Silangcruz JM, et al. A critical review of recent clinical practice guidelines on the diagnosis and treatment of non-neurogenic male lower urinary tract symptoms. Can Urol Assoc J. 2015;9:E463-E70.

[23] Colebatch-Bourn AN, Conaghan PG, Arden NK, Cooper C, Dougados M, Edwards CJ. Raising the quality of rheumatology management recommendations: lessons from the EULAR process 10 years after provision of standard operating procedures. Rheumatology (Oxford). 2015;54:1392-6.

[24] Damiani G, Silvestrini G, Trozzi L, Maci D, Iodice L, Ricciardi W. Quality of dementia clinical guidelines and relevance to the care of older people with comorbidity: evidence from the literature. Clin Interv Aging. 2014;9:1399-407.

[25] Dersch R, Toews I, Sommer H, Rauer S, Meerpohl JJ. Methodological quality of guidelines for management of Lyme neuroborreliosis. BMC Neurol. 2015;15:242.

[26] Don-Wauchope AC, Sievenpiper JL, Hill SA, Iorio A. Applicability of the AGREE II instrument in evaluating the development process and quality of current National Academy of Clinical Biochemistry guidelines. Clin Chem. 2012;58:1426-37.

[27] Falconi M, Crippa S, Chari S, Conlon K, Kim SW, Levy P, et al. Quality assessment of the guidelines on cystic neoplasms of the pancreas. Pancreatology. 2015;15:463-9.

[28] Fisher AR. Development of clinical practice guidelines for urinary continence care of adult stroke survivors in acute and rehabilitation settings. Can J Neurosci Nurs. 2014;36:16-31.

[29] Fouche PF, Zverinova KM. The Australian resuscitation council guideline for managing acute dysrhythmias: an appraisal. Australasian Journal of Paramedicine. 2014;11:article 4.

[30] Gamst-Jensen H, Vedel PN, Lindberg-Larsen VO, Egerod I. Acute pain management in burn patients: appraisal and thematic analysis of four clinical guidelines. Burns. 2014;40:1463-9.

[31] Gandhi S, Verma S, Ethier JL, Simmons C, Burnett H, Alibhai SMH. A systematic review and quality appraisal of international guidelines for early breast cancer systemic therapy: are recommendations sensitive to different global resources? Breast. 2015;24:309-17.

[32] Gillon TE, Pels A, Von Dadelszen P, MacDonell K, Magee LA. Hypertensive disorders of pregnancy: a systematic review of international clinical practice guidelines. PLoS One. 2014;9:e113715.

[33] Girardis M, Cantaroni C, Savoia G, Melotti R, Conti G. A critical appraisal of the quality of analgosedation guidelines in critically ill patients. Minerva Anestesiol. 2016;82:230-5.

[34] Goyet S, Barennes H, Libourel T, Van Griensven J, Frutos R, Tarantola A. Knowledge translation: a case study on pneumonia research and clinical guidelines in a low- income country. Implement Sci. 2014;9:82.

[35] Grimmer K, Dizon JM, Milanese S, King E, Beaton K, Thorpe O, et al. Efficient clinical evaluation of guideline quality: development and testing of a new tool. BMC Med Res Methodol. 2014;14:63.

[36] Gupta M, McCauley J, Farkas A, Gudeloglu A, Neuberger MM, Ho YY, et al. Clinical practice guidelines on prostate cancer: a critical appraisal. J Urol. 2015;193:1153-8.

[37] Gutarra-Vilchez RB, Barajas-Nava L, Aleman A, Sola I, Gich I, Bonfill X, et al. Systematic evaluation of the quality of clinical practice guidelines on the use of assisted reproductive techniques. Hum Fertil. 2014;17:28-36.

[38] Haddadi M, Muhammadnejad S, Sadeghi-Fazel F, Zandieh Z, Rahimi G, Sadighi S, et al. Systematic review of available guidelines on fertility preservation of young patients with breast cancer. Asian Pac J Cancer Prev. 2015;16:1057-62.

[39] Haran C, Van Driel M, Mitchell BL, Brodribb WE. Clinical guidelines for postpartum women and infants in primary care: a systematic review. BMC Pregnancy Childbirth. 2014;14:51.

[40] Harris SR, Schmitz KH, Campbell KL, McNeely ML. Clinical practice guidelines for breast cancer rehabilitation: syntheses of guideline recommendations and qualitative appraisals. Cancer. 2012;118:2312-24.

[41] He Z, Tian H, Song A, Jin L, Zhou X, Liu X, et al. Quality appraisal of clinical practice guidelines on pancreatic cancer: a PRISMA-compliant article. Medicine (Baltimore). 2015;94:e635.

[42] Heine C, O'Halloran R. Central Auditory Processing Disorder: a systematic search and evaluation of clinical practice guidelines. J Eval Clin Pract. 2015;21:988-94.

[43] Henig O, Yahav D, Leibovici L, Paul M. Guidelines for the treatment of pneumonia and urinary tract infections: evaluation of methodological quality using the Appraisal of Guidelines, Research and Evaluation II instrument. Clin Microbiol Infect. 2013;19:1106-14.

[44] Holmer HK, Ogden LA, Burda BU, Norris SL. Quality of clinical practice guidelines for glycemic control in type 2 diabetes mellitus. PLoS One. 2013;8:e58625.

[45] Holvoet T, Raevens S, Vandewynckel YP, Van Biesen W, Geboes K, Van Vlierberghe H. Systematic review of guidelines for management of intermediate hepatocellular carcinoma using the Appraisal of Guidelines Research and Evaluation II instrument. Dig Liver Dis. 2015;47:877-83.

[46] Horner K, O'Malley L, Taylor K, Glenny AM. Guidelines for clinical use of CBCT: a review. Dentomaxillofac Radiol. 2014;44:20140225.

[47] Huang M, Zhou X. Appraisal of guidelines for androgenetic alopecia using the Appraisal of Guidelines for Research and Evaluation II instrument. J Eval Clin Pract. 2015;21:1089-94.

[48] Huang TW, Lai JH, Wu MY, Chen SL, Wu CH, Tam KW. Systematic review of clinical practice guidelines in the diagnosis and management of thyroid nodules and cancer. BMC Med. 2013;11:191.

[49] Jiang Y, Zhu XD, Qu S, Li L, Zhou Z. Guidelines for squamous cell carcinoma of the head and neck: a systematic assessment of quality. Ear Nose Throat J. 2015;94:E14.

[50] Jokhan S, Whitworth MK, Jones F, Saunders A, Heazell AE. Evaluation of the quality of guidelines for the management of reduced fetal movements in UK maternity units. BMC Pregnancy Childbirth. 2015;15:54.

[51] Joosen MC, Brouwers EP, Van Beurden KM, Terluin B, Ruotsalainen JH, Woo JM, et al. An international comparison of occupational health guidelines for the management of mental disorders and stress-related psychological symptoms. Occup Environ Med. 2015;72:313-22.

[52] Kawala C, Fernando D, Tan JK. Quality appraisal of acne clinical practice guidelines, 2008-2013. J Cutan Med Surg. 2014;18:1-7.

[53] Kim SG, Jung HK, Lee HL, Jang JY, Lee H, Kim CG, et al. Guidelines for the diagnosis and treatment of Helicobacter pylori infection in Korea, 2013 revised edition. J Gastroenterol Hepatol. 2014;29:1371-86.

[54] Kirby JS, Scharnitz T, Seiverling EV, Ahrns H, Ferguson S. Actinic keratosis clinical practice guidelines: an appraisal of quality. Dermatology Research and Practice. 2015;2015:456071.

[55] Koh C, Zhao X, Samala N, Sakiani S, Liang TJ, Talwalkar JA. AASLD clinical practice guidelines: a critical review of scientific evidence and evolving recommendations. Hepatology. 2013;58:2142-52.

[56] Kredo T, Gerritsen A, Van Heerden J, Conway S, Siegfried N. Clinical practice guidelines within the Southern African Development Community: a descriptive study of the quality of guideline development and concordance with best evidence for five priority diseases. Health Res Policy Syst. 2012;10:1.

[57] Lambert E, Carey S. Practice guideline recommendations on perioperative fasting: a systematic review. JPEN J Parenter Enteral Nutr. 09.01.2015 [Epub ahead of print].

[58] Langton JM, Drew AK, Mellish L, Olivier J, Ward RL, Pearson SA. The quality of web-based oncology guidelines and protocols: how do international sites stack up? Br J Cancer. 2011;105:1166-72.

[59] Langton JM, Pearson SA. eviQ cancer treatments online: how does the web-based protocol system fare in a comprehensive quality assessment? Asia Pac J Clin Oncol. 2011;7:357-63.

[60] Larmer PJ, Reay ND, Aubert ER, Kersten P. Systematic review of guidelines for the physical management of osteoarthritis. Arch Phys Med Rehabil. 2014;95:375-89.

[61] Lee GY, Yamada J, Kyololo OB, Shorkey A, Stevens B. Pediatric clinical practice guidelines for acute procedural pain: a systematic review. Pediatrics. 2014;133:500-15.

[62] Li CC, Wang YQ, Li YP, Li XL. Critical appraisal of clinical practice guidelines for treating pancreatic cancer based on the global disease burden. J Evid Based Med. 2015;8:11-21.

[63] Li ZY, Luo L, Hu YH, Chen H, Den YK, Tang L, et al. Lung cancer screening: a systematic review of clinical practice guidelines. Int J Clin Pract. 2016;70:20-30.

[64] Loder E, Burch R, Rizzoli P. The 2012 AHS/AAN guidelines for prevention of episodic migraine: a summary and comparison with other recent clinical practice guidelines. Headache. 2012;52:930-45.

[65] Lopez-Vargas PA, Tong A, Sureshkumar P, Johnson DW, Craig JC. Prevention, detection and management of early chronic kidney disease: a systematic review of clinical practice guidelines. Nephrology. 2013;18:592-604.

[66] Luitjes SHE, Wouters MGAJ, Konig T, Hollander KW, Van Os ME, Van Tulder MW, et al. Hypertensive disorders in pregnancy: a review of international guidelines. Hypertens Pregnancy. 2013;32:367-77.

[67] Lytras T, Bonovas S, Chronis C, Konstantinidis AK, Kopsachilis F, Papamichail DP, et al. Occupational asthma guidelines: a systematic quality appraisal using the AGREE II instrument. Occup Environ Med. 2014;71:81-6.

[68] Marciano NJ, Merlin TL, Bessen T, Street JM. To what extent are current guidelines for cutaneous melanoma follow up based on scientific evidence? Int J Clin Pract. 2014;68:761-70.

[69] Nagler EV, Vanmassenhove J, Van der Veer SN, Nistor I, Van Biesen W, Webster AC, et al. Diagnosis and treatment of hyponatremia: a systematic review of clinical practice guidelines and consensus statements. BMC Med. 2014;12:1.

[70] Nelson AE, Allen KD, Golightly YM, Goode AP, Jordan JM. A systematic review of recommendations and guidelines for the management of osteoarthritis: The Chronic Osteoarthritis Management Initiative of the U.S. Bone and Joint Initiative. Semin Arthritis Rheum. 2014;43:701-12.

[71] Norberg MM, Turner MW, Rooke SE, Langton JM, Gates PJ. An evaluation of web-based clinical practice guidelines for managing problems associated with cannabis use. J Med Internet Res. 2012;14:e169.

[72] Nowobilski R, Plaszewski M, Wloch T, Mika P, Gajewski P, Brozek JL. Physiotherapy in asthma: seeking consensus. J Asthma. 2013;50:681-6.

[73] Nuckols TK, Anderson L, Popescu I, Diamant AL, Doyle B, Di Capua P, et al. Opioid prescribing: a systematic review and critical appraisal of guidelines for chronic pain. Ann Intern Med. 2014;160:38-47.

[74] Nuki G. An appraisal of the 2012 American College of Rheumatology guidelines for the management of gout. Curr Opin Rheumatol. 2014;26:152-61.

[75] Olivera MJ, Fory JA, Olivera AJ. Quality assessment of clinical practice guidelines for Chagas disease. Rev Soc Bras Med Trop. 2015;48:343-6.

[76] Padjas A, Kehar R, Aleem S, Mejza F, Bousquet J, Schünemann HJ, et al. Methodological rigor and reporting of clinical practice guidelines in patients with allergic rhinitis: QuGAR study. J Allergy Clin Immunol. 2014;133:777-83.e4.

[77] Pak KJ, Hu T, Fee C, Wang R, Smith M, Bazzano LA. Acute hypertension: a systematic review and appraisal of guidelines. Ochsner J. 2014;14:655-63.

[78] Parisi P, Vanacore N, Belcastro V, Carotenuto M, Del Giudice E, Mariani R, et al. Clinical guidelines in pediatric headache: evaluation of quality using the AGREE II instrument. J Headache Pain. 2014;15:57.

[79] Piano V, Schalkwijk A, Burgers J, Verhagen S, Kress H, Hekster Y, et al. Guidelines for neuropathic pain management in patients with cancer: a European survey and comparison. Pain Practice. 2013;13:349-57.

[80] Polus S, Lerberg P, Vogel J, Watananirun K, Souza JP, Mathai M, et al. Appraisal of WHO guidelines in maternal health using the AGREE II assessment tool. PLoS One. 2012;7:e38891.

[81] Qaseem A, Barry MJ, Denberg TD, Owens DK, Shekelle P. Screening for prostate cancer: a guidance statement from the Clinical Guidelines Committee of the American College of Physicians. Ann Intern Med. 2013;158:761-9.

[82] Rapoport MJ, Weegar K, Kadulina Y, Bedard M, Carr D, Charlton JL, et al. An international study of the quality of national-level guidelines on driving with medical illness. QJM. 2015;108:859-69.

[83] Rios E, Seron P, Lanas F, Bonfill X, Quigley EMM, Alonso-Coello P. Evaluation of the quality of clinical practice guidelines for the management of esophageal or gastric variceal bleeding. Eur J Gastroenterol Hepatol. 2014;26:422-31.

[84] Rohde A, Worrall L, Le Dorze G. Systematic review of the quality of clinical guidelines for aphasia in stroke management. J Eval Clin Pract. 2013;19:994-1003.

[85] Sabharwal S, Gauher S, Kyriacou S, Patel V, Holloway I, Athanasiou T. Quality assessment of guidelines on thromboprophylaxis in orthopaedic surgery. Bone Joint J. 2014;96-B:19-23.

[86] Sabharwal S, Patel NK, Gauher S, Holloway I, Athanasiou T. High methodologic quality but poor applicability: assessment of the AAOS guidelines using the AGREE II instrument. Clin Orthop. 2014;472:1982-8.

[87] San Martin-Galindo L, Rodriguez-Lozano FJ, Abalos-Labruzzi C, Niederman R. European Fissure Sealant Guidelines: assessment using AGREE II. Int J Dent Hyg. 11.09.2015 [Epub ahead of print].

[88] Sanclemente G, Acosta JL, Tamayo ME, Bonfill X, Alonso-Coello P. Clinical practice guidelines for treatment of acne vulgaris: a critical appraisal using the AGREE II instrument. Arch Dermatol Res. 2014;306:269-77.

[89] Santos F, Sola I, Rigau D, Arevalo-Rodriguez I, Seron P, Alonso-Coello P, et al. Quality assessment of clinical practice guidelines for the prescription of antidepressant drugs during pregnancy. Curr Clin Pharmacol. 2012;7:7-14.

[90] Schildmann EK, Schildmann J, Kiesewetter I. Medication and monitoring in palliative sedation therapy: a systematic review and quality assessment of published guidelines. J Pain Symptom Manage. 2015;49:734-46.

[91] Schoenmaker NJ, Tromp WF, Van der Lee JH, Offringa M, Craig JC, Groothoff JW. Quality and consistency of clinical practice guidelines for the management of children on chronic dialysis. Nephrology Dialysis Transplantation. 2013;28:3052-61.

[92] Seron P, Lanas F, Rios E, Bonfill X, Alonso-Coello P. Evaluation of the quality of clinical guidelines for cardiac rehabilitation: a critical review. J Cardiopulm Rehabil Prev. 2014;35:1-12.

[93] Shen J, Sun M, Zhou B, Yan J. Nonconformity in the clinical practice guidelines for subclinical Cushing's syndrome: which guidelines are trustworthy? Eur J Endocrinol. 2014;171:421-31.

[94] Simons JE, Don-Wauchope AC. Evaluation of natriuretic peptide recommendations in heart failure clinical practice guidelines. Clin Biochem. 2016;49:8-15.

[95] Smith CAM, Toupin-April K, Jutai JW, Duffy CM, Rahman P, Cavallo S, et al. A systematic critical appraisal of clinical practice guidelines in juvenile idiopathic arthritis using the appraisal of guidelines for research and evaluation II (AGREE II) instrument. PLoS One. 2015;10:e0137180.

[96] Stacey D, Macartney G, Carley M, Harrison MB, Costars TP. Development and evaluation of evidence-informed clinical nursing protocols for remote assessment, triage and support of cancer treatment-induced symptoms. Nurs Res Pract. 2013;2013:171872.

[97] Syan R, Brucker BM. Guideline of guidelines: urinary incontinence. BJU Int. 2016;117:20-33.

[98] Tian H, Gou Y, Pan Y, Li Q, Wei D, Wang Z, et al. Quality appraisal of clinical practice guidelines on glioma. Neurosurg Rev. 2015;38:39-47.

[99] Tremblay MS, Kho ME, Tricco AC, Duggan M. Process description and evaluation of Canadian physical activity guidelines development. Int J Behav Nutr Phys Act. 2010;7:42.

[100] Tudor KI, Kozina PN, Marusic A. Methodological rigour and transparency of clinical practice guidelines developed by neurology professional societies in Croatia. PLoS One. 2013;8:e69877.

[101] Tunnicliffe DJ, Singh-Grewal D, Kim S, Craig JC, Tong A. Diagnosis, monitoring, and treatment of systemic lupus erythematosus: a systematic review of clinical practice guidelines. Arthritis Care Res. 2015;67:1440-52.

[102] Vanclooster A, Cassiman D, Van Steenbergen W, Swinkels DW, Janssen MCH, Drenth JPH, et al. The quality of hereditary haemochromatosis guidelines: a comparative analysis. Clin Res Hepatol Gastroenterol. 2015;39:205-14.

[103] Vanommeslaeghe F, De Mulder E, Van De Bruaene C, Van De Bruaene L, Lameire N, Van Biesen W. Selecting a strategy for prevention of contrast-induced nephropathy in clinical practice: an evaluation of different clinical practice guidelines using the AGREE tool. Nephrol Dial Transplant. 2015;30:1300-6.

[104] Wang Y, Luo Q, Li Y, Wang H, Deng S, Wei S, et al. Quality assessment of clinical practice guidelines on the treatment of hepatocellular carcinoma or metastatic liver cancer. PLoS One. 2014;9:e103939.

[105] Werner RN, Marinovic B, Rosumeck S, Strohal R, Haering NS, Weberschock T, et al. The quality of European dermatological guidelines: critical appraisal of the quality of EDF guidelines using the AGREE II instrument. J Eur Acad Dermatol Venereol. 2016;30:395-403.

[106] White PE, Shee AW, Finch CF. Independent appraiser assessment of the quality, methodological rigour and transparency of the development of the 2008 international consensus statement on concussion in sport. Br J Sports Med. 2014;48:130-4.

[107] Wilby KJ, Black EK, MacLeod C, Wiens M, Lau TTY, Paiva MA, et al. Critical appraisal of clinical practice guidelines in pediatric infectious diseases. Int J Clin Pharm. 2015;37:799-807.

[108] Wong JJ, Cote P, Shearer HM, Carroll LJ, Yu H, Varatharajan S, et al. Clinical practice guidelines for the management of conditions related to traffic collisions: a systematic review by the OPTIMa Collaboration. Disabil Rehabil. 2015;37:471-89.

[109] Wu AM, Wu CM, Young BK, Wu DJ, Chen A, Margo CE, et al. Evaluation of primary open-angle glaucoma clinical practice guidelines. Can J Ophthalmol. 2015;50:192-6.

[110] Wu AM, Wu CM, Young BK, Wu DJ, Margo CE, Greenberg PB. Critical appraisal of clinical practice guidelines for age-related macular degeneration. J Ophthalmol. 2015;2015:710324.

[111] Wu CM, Wu AM. An evaluation of cataract surgery clinical practice guidelines. Br J Ophthalmol. 2015;99:401-4.

[112] Wu CM, Wu AM, Young BK, Wu DJ, Margo CE, Greenberg PB. An appraisal of clinical practice guidelines for diabetic retinopathy. Am J Med Qual. 05.03.2015 [Epub ahead of print].

[113] Yaman ME, Gudeloglu A, Senturk S, Yaman ND, Tolunay T, Ozturk Y, et al. A critical appraisal of the North American Spine Society guidelines with the Appraisal of Guidelines for Research and Evaluation II instrument. Spine J. 2015;15:777-81.

[114] Yan J, Min J, Zhou B. Diagnosis of pheochromocytoma: a clinical practice guideline appraisal using AGREE II instrument. J Eval Clin Pract. 2013;19:626-32.

[115] Ye ZK, Li C, Zhai SD. Guidelines for therapeutic drug monitoring of vancomycin: a systematic review. PLoS One. 2014;9:e99044.

[116] Yuwen Y, Shi NN, Han XJ, Gao Y, Xu JL, Liu DS, et al. Appraisal of clinical practice guidelines for ischemic stroke management in Chinese medicine with appraisal of guidelines for research and evaluation instrument: a systematic review. Chin J Integr Med. 2015;21:707-15.

[117] Zeng L, Zhang L, Hu Z, Ehle EA, Chen Y, Liu L, et al. Systematic review of evidence-based guidelines on medication therapy for upper respiratory tract infection in children with AGREE instrument. PLoS One. 2014;9:e87711.

[118] Zhang Z, Guo J, Su G, Li J, Wu H, Xie X. Evaluation of the quality of guidelines for myasthenia gravis with the AGREE II instrument. PLoS One. 2014;9:e111796.
