# Supplementary material for: Guideline appraisal with AGREE II: Systematic review of the current evidence on how users handle the 2 overall assessments
Source: PLoS One. 2017 Mar 30;12(3):e0174831. doi: 10.1371/journal.pone.0174831 (PMC5373625; doi:10.1371/journal.pone.0174831)
Supplement: S3 File — (PDF) [file pone.0174831.s003.pdf]

S3: Publications excluded (organized by reasons for exclusion)

No results of appraisal with AGREE II

[1] Aarts MCJ, Van der Heijden GJM, Rovers MM, Grolman W. Remarkable differences between three evidence-based guidelines on management of obstructive sleep apnea-hypopnea syndrome. Laryngoscope. 2013;123:283-91.

[2] Aass Y, McConnell H, Perrier L, Woodbury MG, Sibbald RG. Process for developing evidence-informed practice recommendations: venous leg ulcer example. Advances in Skin & Wound Care. 2009;22:133-40.

[3] Abarshi E, Rietjens J, Caraceni A, Payne S, Deliens L, Van den Block L. Towards a standardised approach for evaluating guidelines and guidance documents on palliative sedation: study protocol. BMC Palliat Care. 2014;13:34.

[4] Aboulsoud S, Huckson S, Wyer P, Lang E. Survey of preferred guideline attributes: what helps to make guidelines more useful for emergency health practitioners? Int J Emerg Med. 2012;5:42.

[5] Ahmed AI, Soliman SM, Awad LA. Validation of evidence-based clinical practice guideline: nursing intervention for newly diagnosed pulmonary tuberculosis patients at community setting. Alexandria Journal of Medicine. 2012;48:155-65.

[6] Alonso-Coello P, Irfan A, Sola I, Gich I, Delgado-Noguera M, Rigau D, et al. The quality of clinical practice guidelines over the last two decades: a systematic review of guideline appraisal studies. Qual Saf Health Care. 2010;19:e58.

[7] Arbour-Nicitopoulos KP, Martin Ginis KA, Latimer-Cheung AE, Bourne C, Campbell D, Cappe S, et al. Development of an evidence-informed leisure time physical activity resource for adults with spinal cord injury: the SCI Get Fit Toolkit. Spinal Cord. 2013;51:491-500.

[8] Armstrong D, Barkun A, Bridges R, Carter R, De Gara C, Dube C, et al. Canadian Association of Gastroenterology consensus guidelines on safety and quality indicators in endoscopy. Can J Gastroenterol. 2012;26:17-31.

[9] Aus G, Chapple C, Hanus T, Irani J, Lobel B, Loch T, et al. TheEuropean Association of Urology (EAU) guidelines methodology: a critical evaluation. Eur Urol. 2009;56:859-64.

[10] Azermai M, Petrovic M, Elseviers MM, Bourgeois J, Van Bortel LM, Van der Stichele RH. Systematic appraisal of dementia guidelines for the management of behavioural and psychological symptoms. Ageing Res Rev. 2012;11:78-86.

[11] Bancos I, Cheng T, Prokop LJ, Montori VM, Murad MH. Endocrine clinical practice guidelines in North America: a systematic assessment of quality. J Clin Epidemiol. 2012;65:520-5.

[12] Barajas-Nava L, Sola I, Delgado-Noguera M, Gich I, Orrego Villagran C, Bonfill X, et al. Quality assessment of clinical practice guidelines in perioperative care: a systematic appraisal. Qual Saf Health Care. 2010;19:e50.

[13] Barclay CA, Vonderhaar KJ, Clark EA. The development of evidence-based care recommendations to improve the safe use of anticoagulants in children. J Pediatr Pharmacol Ther. 2012;17:155-8.

[14] Barkun AN, Bardou M, Kuipers EJ, Sung J, Hunt RH, Martel M, et al. International consensus recommendations on the management of patients with nonvariceal upper gastrointestinal bleeding. Ann Intern Med. 2010;152:101-13.

[15] Batabyal P, Chapman JR, Wong G, Craig JC, Tong A. Clinical practice guidelines on wait-listing for kidney transplantation: consistent and equitable? Transplantation. 2012;94:703-13.

[16] Becker WJ, Findlay T, Moga C, Scott NA, Harstall C, Taenzer P. Guideline for primary care management of headache in adults. Can Fam Physician. 2015;61:670-9.

[17] Bennett WL, Odelola OA, Wilson LM, Bolen S, Selvaraj S, Robinson KA, et al. Evaluation of guideline recommendations on oral medications for type 2 diabetes mellitus: a systematic review. Ann Intern Med. 2012;156:27-36.

[18] Berrigan L, Marshall S, McCullagh S, Velikonja D, Bayley M. Quality of clinical practice guidelines for persons who have sustained mild traumatic brain injury. Brain Inj. 2011;25:742-51.

[19] Bouwmeester W, Van Enst A, Van Tulder M. Quality of low back pain guidelines improved. Spine. 2009;34:2562-7.

[20] Brosseau L. Ottawa panel evidence-based clinical practice guidelines for patient education programmes in the management of osteoarthritis. Health Educ J. 2011;70:318-58.

[21] Brouwers MC, Kho ME, Browman GP, Burgers JS, Cluzeau F, Feder G, et al. Development of the AGREE II, part 1: performance, usefulness and areas for improvement. CMAJ. 2010;182:1045-52.

[22] Brozek J, Jankowski M, Placzkiewicz-Jankowska E, Jaeschke R. International Diabetes Federation document concerning postmeal glycemic control: assessment of quality of clinical practice guidelines using AGREE instrument. Pol Arch Med Wewn. 2009;119:18-24.

[23] Burda BU, Norris SL, Holmer HK, Ogden LA, Smith MEB. Quality varies across clinical practice guidelines for mammography screening in women aged 40-49 years as assessed by AGREE and AMSTAR instruments. J Clin Epidemiol. 2011;64:968-76.

[24] Bush SH, Bruera E, Lawlor PG, Kanji S, Davis DH, Agar M, et al. Clinical practice guidelines for delirium management: potential application in palliative care. J Pain Symptom Manage. 2014;48:249-58.

[25] Canadian Agency for Drugs and Technologies in Health. Quality assessment of the Canadian Thoracic Society guidelines for sleep disordered breathing in adults. Ottawa: CADTH; 2009.

[26] Chen KH, Kao CC, Liu HE, Chiu WT, Kuo KN, Chen CC. Using appraisal of guidelines research and evaluation to appraise nursing clinical practice guidelines in Taiwan and to compare them to international studies. J Exp Clin Med. 2012;4:58-61.

[27] Cheng JS, Lee MJ, Massicotte E, Ashman B, Gruenberg M, Pilcher LE, et al. Clinical guidelines and payer policies on fusion for the treatment of chronic low back pain. Spine. 2011;36:S144-S63.

[28] Chilibeck PD, Vatanparast H, Cornish SM, Abeysekara S, Charlesworth S. Evidence-based risk assessment and recommendations for physical activity: arthritis, osteoporosis, and low back pain. Appl Physiol Nutr Metab. 2011;36:S49-S79.

[29] Cornely OA, Cuenca-Estrella M, Meis JF, Ullmann AJ. European Society of Clinical Microbiology and Infectious Diseases (ESCMID) Fungal Infection Study Group (EFISG) and European Confederation of Medical Mycology (ECMM) 2013 joint guidelines on diagnosis and management of rare and emerging fungal diseases. Clin Microbiol Infect. 2014;20:1-4.

[30] Dagenais S, Tricco AC, Haldeman S. Synthesis of recommendations for the assessment and management of low back pain from recent clinical practice guidelines. Spine J. 2010;10:514-29.

[31] De Boer WE, Bruinvels DJ, Rijkenberg AM, Donceel P, Anema JR. Evidence-based guidelines in the evaluation of work disability: an international survey and a comparison of quality of development. BMC Public Health. 2009;9:349.

[32] De Castro Maymone MB, Gan SD, Bigby M. Evaluating the strength of clinical recommendations in the medical literature: GRADE, SORT, and AGREE. J Invest Dermatol. 2014;134:e25.

[33] De Hert M, Vancampfort D, Correll CU, Mercken V, Peuskens J, Sweers K, et al. Guidelines for screening and monitoring of cardiometabolic risk in schizophrenia: systematic evaluation. Br J Psychiatry. 2011;199:99-105.

[34] De Visschere LMJ, Van der Putten GJ, Vanobbergen JNO, Schols JMGA, De Baat C. An oral health care guideline for institutionalised older people. Gerodontology. 2011;28:307-10.

[35] De Weggheleire A, Bortolotti V, Zolfo M, Crowley S, Colebunders R, Riedner G, et al. Challenges in developing national HIV guidelines: experience from the eastern Mediterranean. Bull World Health Organ. 2011;89:442-50.

[36] Debourdeau P, Beckers M, Gerome P, Durant C, Lacoin Q, Debourdeau A, et al. How to improve the implementation of guidelines on cancer-related thrombosis. Expert Rev Anticancer Ther. 2011;11:473-83.

[37] Delgado-Noguera M, Tort S, Bonfill X, Gich I, Alonso-Coello P. Quality assessment of clinical practice guidelines for the prevention and treatment of childhood overweight and obesity. Eur J Pediatr. 2009;168:789-99.

[38] Dinnes J, Hewison J, Altman DG, Deeks JJ. The basis for monitoring strategies in clinical guidelines: a case study of prostate-specific antigen for monitoring in prostate cancer. CMAJ. 2012;184:169-77.

[39] Ebrahimipour H, Mirfeizi SZ, Najar AV, Kachooei AR, Ariamanesh AS, Ganji R, et al. Developing an appropriateness criteria for knee MRI using the Rand Appropriateness Method (RAM) 2013. Arch Bone Jt Surg. 2014;2:47-51.

[40] Eldh AC, Vogel G, Söderberg A, Blomqvist H, Wengström Y. Use of evidence in clinical guidelines and everyday practice for mechanical ventilation in Swedish intensive care units. Worldviews Evid Based Nurs. 2013;10:198-207.

[41] Espirito Santo A, Choquette A. Experience of adapting and implementing an evidence-based nursing guideline for prevention of diaper dermatitis in a paediatric oncology setting. Int J Evid Based Healthc. 2013;11:121-7.

[42] Ferket BS, Genders TSS, Colkesen EB, Visser JJ, Spronk S, Steyerberg EW, et al. Systematic review of guidelines on imaging of asymptomatic coronary artery disease. J Am Coll Cardiol. 2011;57:1591-600.

[43] Ferket BS, Grootenboer N, Colkesen EB, Visser JJ, Van Sambeek MRHM, Spronk S, et al. Systematic review of guidelines on abdominal aortic aneurysm screening. J Vasc Surg. 2012;55:1296-304.

[44] Ferket BS, Spronk S, Colkesen EB, Hunink MGM. Systematic review of guidelines on peripheral artery disease screening. Am J Med. 2012;125.

[45] Fitzgerald RC, Di Pietro M, Ragunath K, Ang Y, Kang JY, Watson P, et al. British Society of Gastroenterology guidelines on the diagnosis and management of Barrett's oesophagus. Gut. 2014;63:7-42.

[46] Floor-Schreudering A, De Smet PAGM, Buurma H, Amini S, Bouvy ML. Clarity and applicability of drug-drug interaction management guidelines: a systematic appraisal by general practitioners and community pharmacists in the Netherlands. Drug Saf. 2011;34:683-90.

[47] Fortin M, Contant E, Savard C, Hudon C, Poitras ME, Almirall J. Canadian guidelines for clinical practice: an analysis of their quality and relevance to the care of adults with comorbidity. BMC Fam Pract. 2011;12:74.

[48] Foureur M, Ryan CL, Nicholl M, Homer C. Inconsistent evidence: analysis of six national guidelines for vaginal birth after cesarean section. Birth. 2010;37:3-10.

[49] Gaebel W, Riesbeck M, Wobrock T. Schizophrenia guidelines across the world: a selective review and comparison. Int Rev Psychiatry. 2011;23:379-87.

[50] Gallardo CR, Rigau D, Irfan A, Ferrer A, Cayla JA, Bonfill X, et al. Quality of tuberculosis guidelines: urgent need for improvement. Int J Tuberc Lung Dis. 2010;14:1045-51.

[51] Genovesi D, Mazzilli L, Trignani M, Di Tommaso M, Nuzzo A, Biondi E, et al. Developing a decision-making model based on an interdisciplinary oncological care group for the management of colorectal cancer. Anticancer Res. 2014;34:2525-31.

[52] Giannattasio A, Lo Vecchio A, Albano F, Giacomet V, Barbarino A, Guarino A. Flu and pneumococcal immunisations in HIV-infected children: methodological quality of current recommendations. BMJ Qual Saf. 2011;20:432-9.

[53] Ginis KAM, Hicks AL, Latimer AE, Warburton DER, Bourne C, Ditor DS, et al. The development of evidence-informed physical activity guidelines for adults with spinal cord injury. Spinal Cord. 2011;49:1088-96.

[54] Glenny AM, Worthington HV, Clarkson JE, Esposito M. The appraisal of clinical guidelines in dentistry. Eur J Oral Implantol. 2009;2:135-43.

[55] Goergen SK, Rumbold G, Compton G, Harris C. Systematic review of current guidelines, and their evidence base, on risk of lactic acidosis after administration of contrast medium for patients receiving metformin. Radiology. 2010;254:261-9.

[56] Gopalakrishna G, Langendam MW, Scholten RJPM, Bossuyt PMM, Leeflang MMG. Guidelines for guideline developers: a systematic review of grading systems for medical tests. Implement Sci. 2013;8:78.

[57] Gorman SK, Chung MH, Slavik RS, Zed PJ, Wilbur K, Dhingra VK. A critical appraisal of the quality of critical care pharmacotherapy clinical practice guidelines and their strength of recommendations. Intensive Care Med. 2010;36:1636-43.

[58] Green E, Zwaal C, Beals C, Fitzgerald B, Harle I, Jones J, et al. Cancer-related pain management: a report of evidence-based recommendations to guide practice. Clin J Pain. 2010;26:449-62.

[59] Greuter MJE, Van Emmerik NMA, Wouters MGAJ, Van Tulder MW. Quality of guidelines on the management of diabetes in pregnancy: a systematic review. BMC Pregnancy Childbirth. 2012;12:58.

[60] Gupta S, Bhattacharyya OK, Brouwers MC, Estey EA, Harrison MB, Hernandez P, et al. Canadian Thoracic Society: presenting a new process for clinical practice guideline production. Can Respir J. 2009;16:e62-e8.

[61] Häberle J, Huemer M. Evaluation of implementation, adaptation and use of the recently proposed urea cycle disorders guidelines. JIMD Rep. 2015;21:65-70.

[62] Harris MF, Bailey L, Snowdon T, Litt J, Smith JW, Joyner B, et al. Developing the guidelines for preventive care: two decades of experience. Aust Fam Physician. 2010;39:63-5.

[63] Hegarty K, Gunn J, Blashki G, Griffiths F, Dowell T, Kendrick T. How could depression guidelines be made more relevant and applicable to primary care? A quantitative and qualitative review of national guidelines. Br J Gen Pract. 2009;59:e149-e56.

[64] Hester G, Nelson K, Mahant S, Eresuma E, Keren R, Srivastava R. Methodological quality of national guidelines for pediatric inpatient conditions. J Hosp Med. 2014;9:384-90.

[65] Hogeveen SE, Han D, Trudeau-Tavara S, Buck J, Brezden-Masley CB, Quan ML, et al. Comparison of international breast cancer guidelines: are we globally consistent? Cancer guideline AGREEment. Curr Oncol. 2012;19:e184-e90.

[66] Hopayian K, Jackson L. Evaluation of the methodological quality of the Health Protection Agency's 2009 guidance on neuraminidase inhibitors. Qual Prim Care. 2012;20:69-75.

[67] Horiuchi S, Yaju Y, Kataoka Y, Grace Eto H, Matsumoto N. Development of an evidence-based domestic violence guideline: supporting perinatal women-centred care in Japan. Midwifery. 2009;25:72-8.

[68] Hu J, Chen R, Wu S, Tang J, Leng G, Kunnamo I, et al. The quality of clinical practice guidelines in China: a systematic assessment. J Eval Clin Pract. 2013;19:961-7.

[69] Hurkmans EJ, Jones A, Li LC, Vliet Vlieland TPM. Quality appraisal of clinical practice guidelines on the use of physiotherapy in rheumatoid arthritis: a systematic review. Rheumatology (Oxford). 2011;50:1879-88.

[70] Irani S, Rashidian A, Yousefi-Nooraie R, Soltani A. Evaluating clinical practice guidelines developed for the management of thyroid nodules and thyroid cancers and assessing the reliability and validity of the AGREE instrument. J Eval Clin Pract. 2011;17:729-36.

[71] Irving G, Holden J, Edwards J, Reeve J, Dowrick C, Lloyd-Williams M. Chronic heart failure guidelines: do they adequately address patient need at the end-of-life? Int J Cardiol. 2013;168:2304-9.

[72] Jamnik VK, Warburton DER, Makarski J, McKenzie DC, Shephard RJ, Stone JA, et al. Enhancing the effectiveness of clearance for physical activity participation: background and overall process. Appl Physiol Nutr Metab. 2011;36:S3-S13.

[73] Jarrett M. Use of clinical practice guidelines to promote best practice when managing clinical interventions for liver transplant candidates. Prog Transplant. 2009;19:132-40.

[74] Jo MW, Lee JY, Kim NS, Kim SY, Sheen S, Kim SH, et al. Assessment of the quality of clinical practice guidelines in Korea using the AGREE Instrument. J Korean Med Sci. 2013;28:357-65.

[75] Johnstone DE, Buller CE. Pan-Canadian cardiovascular data definitions and quality indicators: a status update. Can J Cardiol. 2012;28:599-601.

[76] Kahn SE, Astles JR, Lo SF, Bennett MJ. The AGREE II instrument is helpful for creation of National Academy of Clinical Biochemistry laboratory medicine practice guidelines. Clin Chem. 2013;59:446-7.

[77] Karamustafalioglu O. Major depressive disorder, mental health care, and the use of guidelines in the Middle East. J Clin Psychiatry. 2010;71:e07.

[78] Kesaniemi A, Riddoch CJ, Reeder B, Blair SN, Sorensen TIA. Advancing the future of physical activity guidelines in Canada: an independent expert panel interpretation of the evidence. Int J Behav Nutr Phys Act. 2010;7:41.

[79] Kinnunen-Amoroso M, Pasternack I, Mattila S, Parantainen A. Evaluation of the practice guidelines of Finnish Institute of Occupational Health with AGREE instrument. Ind Health. 2009;47:689-93.

[80] Kis E, Szegesdi I, Dobos E, Nagy E, Boda K, Kemeny L, et al. Quality assessment of clinical practice guidelines for adaptation in burn injury. Burns. 2010;36:606-15.

[81] Knai C, Brusamento S, Legido-Quigley H, Saliba V, Panteli D, Turk E, et al. Systematic review of the methodological quality of clinical guideline development for the management of chronic disease in Europe. Health Policy. 2012;107:157-67.

[82] La Chapelle CF, Bemelman WA, Rademaker BMP, Van Barneveld TA, Jansen FW. A multidisciplinary evidence-based guideline for minimally invasive surgery part 1: entry techniques and the pneumoperitoneum. Gynecol Surg. 2012;9:271-82.

[83] Latimer-Cheung AE, Martin Ginis KA, Hicks AL, Motl RW, Pilutti LA, Duggan M, et al. Development of evidence-informed physical activity guidelines for adults with multiple sclerosis. Arch Phys Med Rehabil. 2013;94.

[84] Lau CS, Chia F, Harrison A, Hsieh TY, Jain R, Jung SM, et al. APLAR rheumatoid arthritis treatment recommendations. Int J Rheum Dis. 2015;18:685-713.

[85] Lavergne V, Nolin TD, Hoffman RS, Roberts D, Gosselin S, Goldfarb DS, et al. The EXTRIP (EXtracorporeal TReatments In Poisoning) workgroup: guideline methodology. Clin Toxicol. 2012;50:403-13.

[86] Leach MJ, Segal L. Are clinical practical guidelines (CPGs) useful for health services and health workforce planning? A critique of diabetes CPGs. Diabet Med. 2010;27:570-7.

[87] Legido-Quigley H, Panteli D, Brusamento S, Knai C, Saliba V, Turk E, et al. Clinical guidelines in the European Union: mapping the regulatory basis, development, quality control, implementation and evaluation across member states. Health Policy. 2012;107:146-56.

[88] Lepanto L, Tang A, Murphy-Lavallee J, Billiard JS. The Canadian Association of Radiologists guidelines for the prevention of contrast-induced nephropathy: a critical appraisal. Can Assoc Radiol J. 2011;62:238-42.

[89] Levin RF, Ferrara L. Using the Appraisal of Guidelines for Research and Evaluation II to assess clinical practice guidelines. Res Theory Nurs Pract. 2011;25:160-2.

[90] Li-Yu J, Perez EC, Canete A, Bonifacio L, Llamado LQ, Martinez R, et al. Consensus statements on osteoporosis diagnosis, prevention, and management in the Philippines. Int J Rheum Dis. 2011;14:223-38.

[91] Lo Vecchio A, Giannattasio A, Duggan C, De Masi S, Ortisi MT, Parola L, et al. Evaluation of the quality of guidelines for acute gastroenteritis in children with the AGREE instrument. J Pediatr Gastroenterol Nutr. 2011;52:183-9.

[92] Loew L, Brosseau L, Wells GA, Tugwell P, Kenny GP, Reid R, et al. Ottawa panel evidence-based clinical practice guidelines for aerobic walking programs in the management of osteoarthritis. Arch Phys Med Rehabil. 2012;93:1269-85.

[93] Lopez-Medrano F, Cordero E, Gavalda J, Cruzado JM, Marcos MA, Perez-Romero P, et al. Management of influenza infection in solid-organ transplant recipients: consensus statement of the Group for the Study of Infection in Transplant Recipients (GESITRA) of the Spanish Society of Infectious Diseases and Clinical Microbiology (SEIMC) and the Spanish Network for Research in Infectious Diseases (REIPI). Enferm Infecc Microbiol Clin. 2013;31.

[94] Loveday BPT, Srinivasa S, Vather R, Mittal A, Petrov MS, Phillips ARJ, et al. High quantity and variable quality of guidelines for acute pancreatitis: a systematic review. Am J Gastroenterol. 2010;105:1466-76.

[95] Lu Y, Carey S. Translating evidence-based practice guidelines into a summary of recommendations for the nutrition management of upper gastrointestinal cancers. Nutr Clin Pract. 2014;29:518-25.

[96] Mahmud M, Mazza D. Preconception care of women with diabetes: a review of current guideline recommendations. BMC Womens Health. 2010;10:5.

[97] Mar Segui MD, Ronda E, Wimpenny P. Inconsistencies in guidelines for visual health surveillance of VDT workers. J Occup Health. 2012;54:16-24.

[98] Mathur S, Conway DI, Worlledge-Andrew H, Macpherson LMD, Ross AJ. Assessment and prevention of behavioural and social risk factors associated with oral cancer: protocol for a systematic review of clinical guidelines and systematic reviews to inform primary care dental professionals. Syst Rev. 2015;4:184.

[99] Matthews DC. Prevention and treatment of periodontal diseases in primary care. Evid Based Dent. 2014;15:68-9.

[100] Maxwell AP. Diagnosis and management of hyponatraemia: AGREEing the guidelines. BMC Med. 2015;13:31.

[101] McNair RP, Hegarty K. Guidelines for the primary care of lesbian, gay, and bisexual people: a systematic review. Ann Fam Med. 2010;8:533-41.

[102] Meuffels DE, Poldervaart MT, Diercks RL, Fievez AWFM, Patt TW, Van der Hart CP, et al. Guideline on anterior cruciate ligament injury. Acta Orthop. 2012;83:379-86.

[103] Muraro A, Dubois AEJ, Dunngalvin A, Hourihane JO, De Jong NW, Meyer R, et al. EAACI food allergy and anaphylaxis guidelines: food allergy health-related quality of life measures. Allergy. 2014;69:845-53.

[104] Muth C, Gensichen J, Beyer M, Hutchinson A, Gerlach FM. The systematic guideline review: method, rationale, and test on chronic heart failure. BMC Health Serv Res. 2009;9:74.

[105] Nast A, Spuls PH, Ormerod AD, Reytan N, Saiag PH, Smith CH, et al. A critical appraisal of evidence-based guidelines for the treatment of psoriasis vulgaris: 'AGREE-ing' on a common base for European evidence-based psoriasis treatment guidelines. J Eur Acad Dermatol Venereol. 2009;23:782-7.

[106] Ostovar R, Pourreza A, Rashidian A, Rashidi BH, Hantooshzadeh S, Haghollai F, et al. Appropriateness of cesarean sections using the RAND Appropriateness Method criteria. Arch Iran Med. 2012;15:8-13.

[107] Ou Y, Goldberg I, Migdal C, Lee PP. A critical appraisal and comparison of the quality and recommendations of glaucoma clinical practice guidelines. Ophthalmology. 2011;118:1017-23.

[108] Parnell C, Whelton H, O'Mullane D. Water fluoridation. Eur Arch Paediatr Dent. 2009;10:141-8.

[109] Patel ZM, Kennedy DW, Setzen M, Poetker DM, DelGaudio JM. "Sinus headache": rhinogenic headache or migraine? An evidence-based guide to diagnosis and treatment. Int Forum Allergy Rhinol. 2013;3:221-30.

[110] Patel ZM, Setzen M, Sclafani AP, Del Gaudio JM. Concurrent functional endoscopic sinus surgery and septorhinoplasty: using evidence to make clinical decisions. Int Forum Allergy Rhinol. 2013;3:488-92.

[111] Pillastrini P, Gardenghi I, Bonetti F, Capra F, Guccione A, Mugnai R, et al. An updated overview of clinical guidelines for chronic low back pain management in primary care. Joint Bone Spine. 2012;79:176-85.

[112] Poetker DM, Jakubowski LA, Lal D, Hwang PH, Wright ED, Smith TL. Oral corticosteroids in the management of adult chronic rhinosinusitis with and without nasal polyps: an evidence-based review with recommendations. Int Forum Allergy Rhinol. 2013;3:104-20.

[113] Pool F, Goergen S. Quality of the written radiology report: a review of the literature. J Am Coll Radiol. 2010;7:634-43.

[114] Potting C, Mistiaen P, Poot E, Blijlevens N, Donnelly P, Van Achterberg T. A review of quality assessment of the methodology used in guidelines and systematic reviews on oral mucositis. J Clin Nurs. 2009;18:3-12.

[115] Qaseem A, Snow V, Shekelle P, Hopkins R Jr, Owens DK. Screening for HIV in health care settings: a guidance statement from the American College of Physicians and HIV Medicine Association. Ann Intern Med. 2009;150:125-31.

[116] Ramakrishnan VR, Orlandi RR, Citardi MJ, Smith TL, Fried MP, Kingdom TT. The use of image-guided surgery in endoscopic sinus surgery: an evidence-based review with recommendations. Int Forum Allergy Rhinol. 2013;3:236-41.

[117] Ring J, Alomar A, Bieber T, Deleuran M, Fink-Wagner A, Gelmetti C, et al. Guidelines for treatment of atopic eczema (atopic dermatitis) part I. J Eur Acad Dermatol Venereol. 2012;26:1045-60.

[118] Ring J, Alomar A, Bieber T, Deleuran M, Fink-Wagner A, Gelmetti C, et al. Guidelines for treatment of atopic eczema (atopic dermatitis) part II. J Eur Acad Dermatol Venereol. 2012;26:1176-93.

[119] Rodin G, Mackay JA, Zimmermann C, Mayer C, Howell D, Katz M, et al. Clinician-patient communication: a systematic review. Support Care Cancer. 2009;17:627-44.

[120] Rowe RE. Local guidelines for the transfer of women from midwifery unit to obstetric unit during labour in England: a systematic appraisal of their quality. Qual Saf Health Care. 2010;19:90-4.

[121] Rudmik L, Soler ZM, Orlandi RR, Stewart MG, Bhattacharyya N, Kennedy DW, et al. Early postoperative care following endoscopic sinus surgery: an evidence-based review with recommendations. Int Forum Allergy Rhinol. 2011;1:417-30.

[122] Rutter MD, Chattree A, Barbour JA, Thomas-Gibson S, Bhandari P, Saunders BP, et al. British Society of Gastroenterology/Association of Coloproctologists of Great Britain and Ireland guidelines for the management of large non-pedunculated colorectal polyps. Gut. 2015;64:1847-73.

[123] Sampson EL, Van der Steen JT, Pautex S, Svartzman P, Sacchi V, Van den Block L, et al. European palliative care guidelines: how well do they meet the needs of people with impaired cognition? BMJ Support Palliat Care. 2015;5:301-5.

[124] Santaguida PL, Keshavarz H, Carlesso LC, Lomotan M, Gross A, Macdermid JC, et al. A description of the methodology used in an overview of reviews to evaluate evidence on the treatment, harms, diagnosis/classification, prognosis and outcomes used in the management of neck pain. Open Orthop J. 2013;7:461-72.

[125] Schmidt S, Follmann M, Malek N, Manns MP, Greten TF. Critical appraisal of clinical practice guidelines for diagnosis and treatment of hepatocellular carcinoma. J Gastroenterol Hepatol. 2011;26:1779-86.

[126] Schofield P, Sofaer-Bennett B, Hadjistavropoulos T, Zwakhalen S, Brown C, Westerling D, et al. A collaborative expert literature review of pain education, assessment and management. Aging health. 2012;8:43-54.

[127] Seixas M, Weiss M, Muller U. Systematic review of national and international guidelines on attention-deficit hyperactivity disorder. J Psychopharmacol (Oxf). 2012;26:753-65.

[128] Serrano-Aguilar P, Kovacs FM, Cabrera-Hernandez JM, Ramos-Goni JM, Garcia-Perez L. Avoidable costs of physical treatments for chronic back, neck and shoulder pain within the Spanish National Health Service: a cross-sectional study. BMC Musculoskelet Disord. 2011;12:287.

[129] Sevrain M, Villani AP, Rouzaud M, Barnetche T, Paul C, Richard MA, et al. Treatment (biotherapy excluded) of psoriatic arthritis: an appraisal of methodological quality of international guidelines. J Eur Acad Dermatol Venereol. 2014;28:33-9.

[130] Shah P, Moles DR, Parekh S, Ashley P, Siddik D. Evaluation of pediatric dentistry guidelines using the AGREE instrument. Pediatr Dent. 2011;33:120-9.

[131] Shimbo T, Fukui T, Ishioka C, Okamoto K, Okamoto T, Kameoka S, et al. Quality of guideline development assessed by the Evaluation Committee of the Japan Society of Clinical Oncology. Int J Clin Oncol. 2010;15:227-33.

[132] Shinohara Y, Nagayama M, Origasa H. Postpublication external review of the Japanese guidelines for the management of stroke 2004. Stroke. 2009;40:1439-43.

[133] Simone B, De Feo E, Nicolotti N, Ricciardi W, Boccia S. Methodological quality of English-language genetic guidelines on hereditary breast-cancer screening and management: an evaluation using the AGREE instrument. BMC Med. 2012;10:143.

[134] Simone B, De Feo E, Nicolotti N, Ricciardi W, Boccia S. Quality evaluation of guidelines on genetic screening, surveillance and management of hereditary colorectal cancer. Eur J Public Health. 2012;22:914-20.

[135] Simons MP, Aufenacker T, Bay-Nielsen M, Bouillot JL, Campanelli G, Conze J, et al. European Hernia Society guidelines on the treatment of inguinal hernia in adult patients. Hernia. 2009;13:343-403.

[136] Soler ZM, Oyer SL, Kern RC, Senior BA, Kountakis SE, Marple BF, et al. Antimicrobials and chronic rhinosinusitis with or without polyposis in adults: an evidenced-based review with recommendations. Int Forum Allergy Rhinol. 2013;3:31-47.

[137] Spence K, Henderson-Smart D, New K, Evans C, Whitelaw J, Woolnough R. Evidenced-based clinical practice guideline for management of newborn pain. J Paediatr Child Health. 2010;46:184-92.

[138] Spuls PI, Nast A. Evaluation of and perspectives on guidelines: what is important? J Invest Dermatol. 2010;130:2348-9.

[139] Stein DJ, Ipser J, McAnda N. Pharmacotherapy of posttraumatic stress disorder: a review of meta-analyses and treatment guidelines. CNS Spectr. 2009;14:25-31.

[140] Stergiou-Kita M, Dawson D, Rappolt S. Inter-professional clinical practice guideline for vocational evaluation following traumatic brain injury: a systematic and evidence-based approach. J Occup Rehabil. 2012;22:166-81.

[141] Stergiou-Kita M, Grigorovich A, Gomez M. Development of an inter-professional clinical practice guideline for vocational evaluation following severe burn. Burns. 2014;40:1149-63.

[142] Stoffer MA, Smolen JS, Woolf A, Ambrozic A, Bosworth A, Carmona L, et al. Development of patient-centred standards of care for rheumatoid arthritis in Europe: the eumusc.net project. Ann Rheum Dis. 2014;73:902-5.

[143] Stone MA, Wilkinson JC, Charpentier G, Clochard N, Grassi G, Lindblad U, et al. Evaluation and comparison of guidelines for the management of people with type 2 diabetes from eight European countries. Diabetes Res Clin Pract. 2010;87:252-60.

[144] Strech D, Schildmann J. Quality of ethical guidelines and ethical content in clinical guidelines: the example of end-of-life decision-making. J Med Ethics. 2011;37:390-6.

[145] Takeuchi H, Saeki T, Aiba K, Tamura K, Aogi K, Eguchi K, et al. Japanese Society of Clinical Oncology clinical practice guidelines 2010 for antiemesis in oncology: executive summary. Int J Clin Oncol. 2016;21:1-12.

[146] Tan JKL, Wolfe BJ, Bulatovic R, Jones EB, Lo AY. Critical appraisal of quality of clinical practice guidelines for treatment of psoriasis vulgaris, 2006-2009. J Invest Dermatol. 2010;130:2389-95.

[147] Tavender EJ, Bosch M, Green S, O'Connor D, Pitt V, Phillips K, et al. Quality and consistency of guidelines for the management of mild traumatic brain injury in the emergency department. Acad Emerg Med. 2011;18:880-9.

[148] Thomas SG, Goodman JM, Burr JF. Evidence-based risk assessment and recommendations for physical activity clearance: established cardiovascular disease. Appl Physiol Nutr Metab. 2011;36:S190-S213.

[149] Tong A, Chapman JR, Wong G, De Bruijn J, Craig JC. Screening and follow-up of living kidney donors: a systematic review of clinical practice guidelines. Transplantation. 2011;92:962-72.

[150] Tremblay MS, Leblanc AG, Carson V, Choquette L, Connor Gorber S, Dillman C, et al. Canadian physical activity guidelines for the early years (aged 0-4 years). Appl Physiol Nutr Metab. 2012;37:345-69.

[151] Tremblay MS, Leblanc AG, Carson V, Choquette L, Connor Gorber S, Dillman C, et al. Canadian sedentary behaviour guidelines for the early years (aged 0-4 years). Appl Physiol Nutr Metab. 2012;37:370-91.

[152] Tremblay MS, Leblanc AG, Janssen I, Kho ME, Hicks A, Murumets K, et al. Canadian sedentary behaviour guidelines for children and youth. Appl Physiol Nutr Metab. 2011;36:59-64.

[153] Tremblay MS, Warburton DER, Janssen I, Paterson DH, Latimer AE, Rhodes RE, et al. New Canadian physical activity guidelines. Appl Physiol Nutr Metab. 2011;36:36-46.

[154] Tripathi D, Stanley AJ, Hayes PC, Patch D, Millson C, Mehrzad H, et al. U.K. guidelines on the management of variceal haemorrhage in cirrhotic patients. Gut. 2015;64:1680-704.

[155] Tugwell P, Pottie K, Welch V, Ueffing E, Chambers A, Feightner J. Evaluation of evidence-based literature and formulation of recommendations for the clinical preventive guidelines for immigrants and refugees in Canada. CMAJ. 2011;183:E933-E8.

[156] Ullmann AJ, Cornely OA, Donnelly JP, Akova M, Arendrup MC, Arikan-Akdagli S, et al. ESCMID* guideline for the diagnosis and management of Candida diseases 2012: developing European guidelines in clinical microbiology and infectious diseases. Clin Microbiol Infect. 2012;18:1-8.

[157] Unden J, Ingebrigtsen T, Romner B. Scandinavian guidelines for initial management of minimal, mild and moderate head injuries in adults: an evidence and consensus-based update. BMC Med. 2013;11:50.

[158] Van de Bovenkamp HM, Zuiderent-Jerak T. An empirical study of patient participation in guideline development: exploring the potential for articulating patient knowledge in evidence-based epistemic settings. Health Expect. 03.05.2013 [Epub ahead of print].

[159] Van de Velde S, Heselmans A, Donceel P, Vandekerckhove P, Ramaekers D, Aertgeerts B. Rigour of development does not AGREE with recommendations in practice guidelines on the use of ice for acute ankle sprains. BMJ Qual Saf. 2011;20:747-55.

[160] Van den Berg T, Engelhardt EG, Haanstra TM, Langius JAE, Van Tulder MW. Methodology of clinical nutrition guidelines for adult cancer patients: how good are they according to AGREE criteria? JPEN J Parenter Enteral Nutr. 2012;36:316-22.

[161] Van Diermen DE, Aartman IHA, Baart JA, Hoogstraten J, Van der Waal I. Dental management of patients using antithrombotic drugs: critical appraisal of existing guidelines. Oral Surg Oral Med Oral Pathol Oral Radiol Endod. 2009;107:616-24.

[162] Vancampfort D, Sweers K, Probst M, Mitchell AJ, Knapen J, De Hert M. Quality assessment of physical activity recommendations within clinical practice guidelines for the prevention and treatment of cardio-metabolic risk factors in people with schizophrenia. Community Ment Health J. 2011;47:703-10.

[163] Vasse E, Vernooij-Dassen M, Cantegreil I, Franco M, Dorenlot P, Woods B, et al. Guidelines for psychosocial interventions in dementia care: a European survey and comparison. Int J Geriatr Psychiatry. 2012;27:40-8.

[164] Warburton DER, Jamnik VK, Bredin SSD, McKenzie DC, Stone J, Shephard RJ, et al. Evidence-based risk assessment and recommendations for physical activity clearance: an introduction. Appl Physiol Nutr Metab. 2011;36:S1-S2.

[165] Warriner RA 3rd, Carter MJ. The current state of evidence-based protocols in wound care. Plast Reconstr Surg. 2011;127:144S-53S.

[166] Wens J, Van Royen P. Comment on: "Evaluation and comparison of guidelines for the management of people with type 2 diabetes from eight European countries" by Stone et al. on behalf of the GUIDANCE study group (Diabetes Res Clin Pract 2010; 87(2): 252-60). Diabetes Res Clin Pract. 2011;92:407-8.

[167] Wijkstra J, Schubart CD, Nolen WA. Treatment of unipolar psychotic depression: the use of evidence in practice guidelines. World J Biol Psychiatry. 2009;10:409-15.

[168] Wilson DC, Thomas AG, Croft NM, Newby E, Akobeng AK, Sawczenko A, et al. Systematic review of the evidence base for the medical treatment of paediatric inflammatory bowel disease. J Pediatr Gastroenterol Nutr. 2010;50:S14-S34.

[169] Wilson MG, Dickie M, Cooper CL, Carvalhal A, Bacon J, Rourke SB. Treatment, care and support for people co-infected with HIV and hepatitis C: a scoping review. Open Med. 2009;3:e184-95.

[170] Wilting I, Egberts ACG, Heerdink ER, Ververs TFT, Meulenbelt J, Nolen WA. Evaluation of available treatment guidelines for the management of lithium intoxication. Ther Drug Monit. 2009;31:247-60.

[171] Winther LP, Mitchell AU, Moller AM. Inconsistencies in clinical guidelines for obstetric anaesthesia for Caesarean section: a comparison of the Danish, English, American, and German guidelines with regard to developmental quality and guideline content. Acta Anaesthesiol Scand. 2013;57:141-9.

[172] Wiseman R, Cohen K, Gray A, Jamaloodien K, Kredo T, Miot J, et al. AGREE to disagree: critical appraisal and the publication of practice guidelines. S Afr Med J. 2014;104:345-6.

[173] Wright F, Spithoff K, Easson A, Murray C, Toye J, McCready D, et al. Primary excision margins and sentinel lymph node biopsy in clinically node-negative melanoma of the trunk or extremities. Clin Oncol (R Coll Radiol). 2011;23:572-8.

[174] Yu WY, Xu JL, Shi NN, Wang LY, Han XJ, Wang YY, et al. Assessing the quality of the first batch of evidence-based clinical practice guidelines in traditional Chinese medicine. J Tradit Chin Med. 2011;31:376-81.

[175] Zadvinskis IM, Grudell BA. Clinical practice guideline appraisal using the AGREE instrument: renal screening. Clin Nurse Spec. 2010;24:209-14.

[176] Zheng ZH, Cui SQ, Lu XQ, Zakus D, Liang WN, Huang F, et al. Analysis of the status of Chinese clinical practice guidelines development. BMC Health Serv Res. 2012;12:218.

No reporting of standardized domain scores

[1] Abrahamyan L, Boom N, Donovan LR, Tu JV. An international environmental scan of quality indicators for cardiovascular care. Can J Cardiol. 2012;28:110-8.

[2] Brenol CV, Nava JIG, Soriano ER. Proper management of rheumatoid arthritis in Latin America. What the guidelines say? Clin Rheumatol. 2015;34:S51-S5.

[3] Brouwers MC, Kho ME, Browman GP, Burgers JS, Cluzeau F, Feder G, et al. The Global Rating Scale complements the AGREE II in advancing the quality of practice guidelines. J Clin Epidemiol. 2012;65:526-34.

[4] Brouwers MC, Rawski E, Spithoff K, Oliver TK. Inventory of Cancer Guidelines: a tool to advance the guideline enterprise and improve the uptake of evidence. Expert Rev Pharmacoecon Outcomes Res. 2011;11:151-61.

[5] Chen YL, Yao L, Xiao XJ, Wang Q, Wang ZH, Liang FX, et al. Quality assessment of clinical guidelines in China: 1993-2010. Chin Med J (Engl). 2012;125:3660-4.

[6] Conway MA, McCollom C, Bannon C. Central venous catheter flushing recommendations: a systematic evidence-based practice review. J Pediatr Oncol Nurs. 2014;31:185-90.

[7] Edstrom E, Westerberg L, Henricson M. Appraisal of guidelines for pre-operative body wash. Br J Nurs. 2014;23:1106-13.

[8] Gagliardi AR, Brouwers MC. Do guidelines offer implementation advice to target users? A systematic review of guideline applicability. BMJ Open. 2015;5:e007047.

[9] Guo J, Cheng C, Yan W, Xu G, Feng J, Wang T, et al. Systematic review of clinical practice guidelines related to multiple sclerosis. PLoS One. 2014;9:e106762.

[10] Hazlewood GS, Akhavan P, Schieir O, Marshall D, Tomlinson G, Bykerk V, et al. Adding a "GRADE" to the quality appraisal of rheumatoid arthritis guidelines identifies limitations beyond AGREE-II. J Clin Epidemiol. 15.09.2014 [Epub ahead of print].

[11] Isaac A, Saginur M, Hartling L, Robinson JL. Quality of reporting and evidence in American Academy of Pediatrics guidelines. Pediatrics. 2013;131:732-8.

[12] Jacobs C, Graham ID, Makarski J, Chasse M, Fergusson D, Hutton B, et al. Clinical practice guidelines and consensus statements in oncology: an assessment of their methodological quality. PLoS One. 2014;9:e110469.

[13] Jakes AD, Marec-Berard P, Phillips RS, Stark DP. Critical review of clinical practice guidelines for fertility preservation in teenagers and young adults with cancer. J Adolesc Young Adult Oncol. 2014;3:144-52.

[14] Jiang M, Liao LY, Liu XQ, He WQ, Guan WJ, Chen H, et al. Quality assessment of clinical practice guidelines for respiratory diseases in China: a systematic appraisal. Chest. 2015;148:759-66.

[15] Kaufman AL, Spitz J, Jacobs M, Sorrentino M, Yuen S, Danahey K, et al. Evidence for clinical implementation of pharmacogenomics in cardiac drugs. Mayo Clin Proc. 2015;90:716-29.

[16] Knops RR, Kremer LC, Verhagen AA. Paediatric palliative care: recommendations for treatment of symptoms in the Netherlands. BMC Palliat Care. 2015;14:57.

[17] Lee YK, Shin ES, Shim JY, Min KJ, Kim JM, Lee SH. Developing a scoring guide for the Appraisal of Guidelines for Research and Evaluation II instrument in Korea: a modified Delphi consensus process. J Korean Med Sci. 2013;28:190-4.

[18] Malheiro R, De Monteiro-Soares M, Hassan C, Dinis-Ribeiro M. Methodological quality of guidelines in gastroenterology. Endoscopy. 2014;46:513-25.

[19] Ngo J, Holroyd-Leduc JM. Systematic review of recent dementia practice guidelines. Age Ageing. 2015;44:25-33.

[20] Oh MK, Jo H. Improving the reliability of clinical practice guideline appraisals: effects of the Korean AGREE II scoring guide. J Korean Med Sci. 2014;29:771-5.

[21] Petrarca S, Follmann M, Breitbart EW, Nolte S. Critical appraisal of clinical practice guidelines for adaptation in the evidence-based guideline "prevention of skin cancer". JAMA Dermatol. 2013;149:466-71.

[22] Rashidian A, Yousefi-Nooraie R. Development of a Farsi translation of the AGREE instrument, and the effects of group discussion on improving the reliability of the scores. J Eval Clin Pract. 2012;18:676-81.

[23] Rosumeck S, Sporbeck B, Rzany B, Nast A. Disclosure of potential conflicts of interest in dermatological guidelines in Germany: an analysis; status quo and quo vadis. J Dtsch Dermatol Ges. 2011;9:297-304.

[24] Sabharwal S, Patel V, Nijjer SS, Kirresh A, Darzi A, Chambers JC, et al. Guidelines in cardiac clinical practice: evaluation of their methodological quality using the AGREE II instrument. J R Soc Med. 2013;106:315-22.

[25] Shah A, Mohamed-Ahmed O, Peirsegaele P, McClymont C, Knight M. Incident reviews in UK maternity units: a systematic appraisal of the quality of local guidelines. BMC Pregnancy Childbirth. 2015;15:58.

[26] Shawyer AC, Livingston MH, Manja V, Brouwers MC. The quality of guidelines in pediatric surgery: can we all AGREE? Pediatr Surg Int. 2015;31:61-8.

[27] Siering U, Ruther A. Erfahrungen mit Leitliniensynopsen am Beispiel der Synopsen für Disease-Management-Programme (DMP). Z Evid Fortbild Qual Gesundhwes. 2014;108:560-8.

[28] Sonawane DB, Karvande SS, Cluzeau FA, Chavan SA, Mistry NF. Appraisal of maternity management and family planning guidelines using the agree II instrument in India. Indian J Public Health. 2015;59:264-71.

[29] Sun M, Zhang M, Shen J, Yan J, Zhou B. Critical appraisal of international guidelines for the management of diabetic neuropathy: is there global agreement in the internet era? Int J Endocrinol. 2015;2015:519032.

[30] Tirotta D, Marchetti A, Di Lillo M, Pomero F, Re R, Meschi M, et al. Abdominal pain: a synthesis of recommendations for its correct management. Italian Journal of Medicine. 2015;9:193-202.

[31] Van Biesen W, Van de Velde T, Slabbaert M, Simoens I, Van Paemel R, Van der Veer SN. Blood pressure management in patients with chronic kidney disease: an appraisal and summary of existing guidelines. Acta Clin Belg. 2013;68:394-8.

[32] Yuwen Y, Han X, Shi N, Wang L, Liao X, Zhong L, et al. Appraisal of clinical practice guidelines for the management of rheumatoid arthritis in traditional Chinese medicine using the AGREE II instrument: a systematic review. Eur J Integr Med. 2014;6:176-85.

[33] Yuwen Y, Shi NN, Han XJ, Gao Y, Xu JL, Liu DS, et al. Appraisal of clinical practice guidelines for ischemic stroke management in Chinese medicine with appraisal of guidelines for research and evaluation instrument: a systematic review. Chin J Integr Med. 18.06.2014 [Epub ahead of print].

[34] Zargham-Boroujeni A, Zoafa A, Marofi M, Badiee Z. Compilation of the neonatal palliative care clinical guideline in neonatal intensive care unit. Iran J Nurs Midwifery Res. 2015;20:309-14.

[35] Zhang ZW, Liu XW, Xu BC, Wang SY, Li L, Kang YY, et al. Analysis of quality of clinical practice guidelines for otorhinolaryngology in China. PLoS One. 2013;8:e53566.

Published before 2009

None

Full text not available

[1] Abstracts of 16th National Congress of SIGENP. Dig Liver Dis. 2009;41:S199-S244.

[2] Abarshi E, Rietjens J, Caraceni A, Payne S, Deliens L, Van den Block L. International comparison of content and quality of clinical practice guidelines on palliative sedation. Palliat Med. 2014;28:571.

[3] Abarshi E, Rietjens J, Caraceni A, Payne S, Deliens L, Van den Block L. A standardised approach for comparing guidance documents on sedation practices in palliative care. Palliat Med. 2014;28:659.

[4] Abdelsattar ZM, Wong SL. A comparison of rectal cancer clinical practice guidelines. Ann Surg Oncol. 2014;21:S14.

[5] Agrawal SG. ECIL vs. IDSA guidelines. Mycoses. 2011;54:41-2.

[6] Ahmed A, Soliman S, Awad LA. A validated clinical practice guideline for community health nurses working in tuberculosis out-patient clinics. Int J Infect Dis. 2010;14:e147.

[7] Akhavan P, Schieir O, Hazlewood G, Bykerk V, Bombardier C. Strength of evidence in guidelines regarding the pharmacological management of RA. J Rheumatol. 2010;37:1314.

[8] Al-Mustafa B, Al-Faraj N, Al-Eid N, Al-Mobarak A, Hejlis M, Al-Salman E, et al. Cardiometabolic risk management guidelines in primary care: addressing barriers of implementation. J Hypertens. 2012;30:e138-e9.

[9] Azermai M, Petrovic M, Elseviers M, Bourgois J, Van Bortel L, Van der Stichele R. Systematic quality appraisal and recommendation extraction from dementia guidelines on management of behavioural and psychological symptoms. Basic Clin Pharmacol Toxicol. 2011;109:94-5.

[10] Banaschewski T, Hohmann S, Taylor E, Coghill D, Zuddas A, Soutullo C, et al. ESCAP guidelines workshop ADHD. Eur Child Adolesc Psychiatry. 2013;22:S114.

[11] Bancos I, Cheng T, Prokop L, Montori V, Murad M. Should you follow practice guidelines in endocrinology? A systematic assessment of their methodological quality. Endocr Rev. 2011;32:Abstract P2-765.

[12] Barrie A, Bedford M, Elfvinge P, Gronhaug S, Mueller-Kaegi E, Schrijvers L. Evidence-based development of clinical practice guideline for peripheral self-infusion of prophylaxis. Haemophilia. 2015;21:65-6.

[13] Barriocanal A, Lopez A, Costa J, Montane E. Quality of clinical practice guidelines for peripheral arterial disease: a systematic review. Basic Clin Pharmacol Toxicol. 2013;113:11.

[14] Batabyal P, Chapman JR, Wong G, Craig J, Tong A. Clinical practices guidelines on waitlisting for kidney transplantation: a systematic review. Transplantation. 2012;94:525.

[15] Batabyal P, Chapman JR, Wong G, Craig JC, Tong A. Clinical practice guidelines on waitlisting for kidney transplantation: consistent and equitable? Nephrology. 2012;17:82.

[16] Bennett WL, Odelola O, Wilson L, Bolen S, Bass EB, Dalal D, et al. Systematic review of clinical practice guidelines on the pharmacologic treatment of type 2 diabetes mellitus: are guidelines evidence-based? J Gen Intern Med. 2011;26:S70-S1.

[17] Binepal N, Moore G, Lemyre B, Daboval T, Dunn S, Leduc S. Quality appraisal of international guidelines on perinatal care of extremely premature infants. Arch Dis Child. 2014;99:A129-A30.

[18] Bourn AN, Conaghan PG, Arden NK, Cooper C, Dougados M, Edwards CJ. The quality of eular management recommendations: a review ten years after publication of standardised operating procedures. Ann Rheum Dis. 2014;73:173.

[19] Brener SS, Nikitovic M, Chambers A, Ghazipura M, Schaink AK, Lambrinos AI, et al. Evidence-based practice recommendations: Health Quality Ontario's approach. Value Health. 2014;17:A29.

[20] Brosseau L, Toupin-April K, Paterson G, De Angelis G, Loew L. The non pharmacological management of osteoarthritis & rheumatoid arthritis. J Rheumatol. 2014;41:1520-1.

[21] Brouwers MC, Rawski E, Bahirathan L, Spithoff K, Zwaal C. SAGE directory of cancer guidelines. J Clin Oncol. 2012;30:Abstract 306.

[22] Brunetti VI, Centeno P, Monteagudo EG, Anchorena RM. Evidence-based guideline for the hormone modulation of the adult brain-dead organ donor: vasopressin, corticosteroids and thyroid hormones use. Transplantation. 2013;96:S221.

[23] Butcher R, Lin F, Jones J, Rickard C. The quality of published central venous catheter clinical practice guidelines: a systematic appraisal using the AGREE II instrument. J Vasc Access. 2014;15:208.

[24] Cammarata S, Adami S, Paciello A, Del Santo F, Simbula S, Messori A. IPERPTO: a new idea for the online hospital drug formulary. Eur J Hosp Pharm Sci Pract. 2012;19:200-1.

[25] Choi J, Choi TY, Lee JA, Jun JH, Lee MS. The quality of clinical practice guidelines in traditional medicine in Korea: appraisal using the AGREE II instrument. Integrative Medicine Research. 2015;4:135.

[26] Currie A, Malik A, Askari A, Nachiappan S, Thomas-Gibson S, Faiz O, et al. An international comparison of clinical practice guidelines for the management of malignant colorectal polyps. Gut. 2015;64:A332.

[27] De Hert M. Systematic evaluation of guidelines for monitoring cardio-metabolic risk in schizophrenia. Schizophr Res. 2012;136:S59.

[28] De Hert M, Vancampfort D, Correll C, Peuskens J, Van Winkel R, Mitchell A. A systematic evaluation and comparison of the guidelines for screening and monitoring of cardiometabolic risk in people with schizophrenia. Eur Psychiatry. 2011;26:2189.

[29] De Seixas MD, Muller U. Systematic review of national and international guidelines on ADHD. Eur Psychiatry. 2009;24:S395.

[30] Deutsch L, King E, Sinclair S, Hastings P, Mazza D, Chakraborty S, et al. Investigating symptoms of lung cancer: an evidence-based guide for general practitioners. J Thorac Oncol. 2013;8:S980-S1.

[31] Gaebel W. Recommendations on pharmacological treatment: recent updates. Eur Arch Psychiatry Clin Neurosci. 2011;261:S49.

[32] Gaitan Duarte H, Rodriguez A, Angel-Muller E, Lopez-Gomez H, Estrada S, Cinets A. Clinical practice guideline for sexually transmitted infections and other infection of the genital tract: syndromic management; 2012. Sex Transm Infect. 2013;89:A368.

[33] Gou Y, Li F, Jin A, Wang W, Huang X. An appraisal of clinical practice guidelines for irritable bowel syndrome. Journal of Gastroenterology and Hepatology (Australia). 2015;30:44.

[34] Harle I, Saunders D, Bak K, Jarvis V, Argier P, Cheung A, et al. Development of a multidisciplinary, evidence based symptom management tool for oral care in cancer therapy. Support Care Cancer. 2013;21:S151.

[35] Harstall C. Generating the evidence for the development of guideline for headache disorders. Pain Res Manag. 2012;17:202.

[36] Hazlewood G, Schieir O, Akhavan P, Bykerk V, Bombardier C. Finding guidelines to AGREE on: a quality appraisal of international guidelines on the pharmacological teatment of rheumatoid arthritis. J Rheumatol. 2010;37:1314.

[37] Hennessy K, Woodburn J, Steultjens M. Critical appraisal of clinical practice guidelines for foot and ankle management in rheumatoid arthritis. Rheumatology (Oxford). 2013;52:i108.

[38] Jacobs CM, Graham ID, Makarski J, Chasse M, Fergusson D, Clemons MJ. An evaluation of the methodologic quality of clinical practice guidelines and consensus statements in oncology. J Clin Oncol. 2014;32:Abstract e17629.

[39] Jiang M, Li YM, Zheng JP. Quality assessment of clinical practice guidelines inrespiratory diseases in China: a systematic appraisal. Respirology. 2014;19:195.

[40] Jiang M, Liao L, Liu X, Guan W, Li Y. Quality assessment of clinical practice guidelines for respiratory diseases in China: a systematic appraisal. Eur Respir J. 2015;46:PA711.

[41] Keating D, McWilliams S, Cousins G, Strawbridge J, Clarke M. A systematic review of international guidelines for the pharmacological treatment of first episode schizophrenia. Early Intervention in Psychiatry. 2014;8:78.

[42] Kim HY, Lee H, Kim HK, Park HK, Oh MG, Park JW. Appraisal of the guidelines on the management of hepatocellular carcinoma. Hepatology. 2011;54:1399A-400A.

[43] Kirby J, Scharnitz TP, Seiverling ES, Ahrns HS, Ferguson SS. Evaluation of actinic keratosis clinical practice guidelines. J Invest Dermatol. 2015;135:S51.

[44] Kis E, Brychta P. Methodological background of the European guidelines for burn care practice. Burns. 2011;37:S24.

[45] Kobleder A, Ivanovic N, Raphaelis S, Gafner D, Senn B. Health education in patients with vulvar neoplasia and surgical treatment: an evidence based guideline. Int J Gynecol Cancer. 2014;24:1131-2.

[46] La Chapelle C, Jansen FW. Multidisciplinary guideline development in MIS: a challenge for all? Gynecol Surg. 2011;8:S66-S7.

[47] Lalucat-Jo L, Carbonero M, Faus G, Guillamon I, Martinez C, Laia M, et al. Comparative study of international clinical practice guidelines about schizophrenia and early psychosis management. Eur Arch Psychiatry Clin Neurosci. 2009;259:S80-S1.

[48] Lamontagne ME, Swaine B, St-Pierre C, Truchon C. A scoping review and a quality evaluation of clinical practice guidelines for the rehabilitation of adults with moderate-tosevere traumatic brain injury. Brain Inj. 2014;28:827.

[49] Larenas-Linnemann D. ARIA Mexico 2014: transculturization of a guideline involving 11 national medical societies. World Allergy Organ J. 2014;8:A286.

[50] Lee T, Park B, Bolla KC, Sung J, Chiluveru S, Park S, et al. Systematic quality assessment of published antishivering protocols. Neurocrit Care. 2014;21:S77.

[51] Li-Yu JT, Perez EC, Canete A, Bonifacio L, Llamado LQ, Martinez R, et al. Consensus guidelines on osteoporosis diagnosis, prevention, and management in the Philippines. Osteoporos Int. 2010;21:S751.

[52] Lo Vecchio A, Giannattasio A, De Masi S, Ortisi MT, Parola L, Duggan C, et al. Evaluation of quality of guidelines for acute gastroenteritis in children with the AGREE instrument. Dig Liver Dis. 2009;41:S202.

[53] Lo Vecchio A, Giannattasio A, Duggan C, De Masi S, Ortisi M, Parola L, et al. Evaluation of the quality of guidelines for acute gastroenteritis in children, with the AGREE instrument. J Pediatr Gastroenterol Nutr. 2010;50:E27.

[54] McCaul M. Spinal clearance guideline for out-of hospital providers. Afr J Emerg Med. 2013;3:S7.

[55] McGillion M. The new CPS development and review process. Pain Res Manag. 2013;18:e14.

[56] Mei J. Cough clinical practice guidelines: a critical review of scientific evidence and recommendations. Respirology. 2015;20:105.

[57] Mohamed S, Al-Juryyan N, Babiker A, Al-Otibi H, Hasanato R, Omer H, et al. Development and implementation of clinical practice guidelines in diabetic ketoacidosis: NICE is also nice in the Middle East. Horm Res Paediatr. 2013;80:290-1.

[58] Mohamed S, Al-Juryyan N, Babiker A, Iqbal SM, Osman MEF, Al-Nemri A. Development and implementation of clinical practice guidelines in pediatric endocrinology: challenges and opportunities. Endocr Rev. 2013;34:Abstract MON-282.

[59] Moore G, Lemyre B, Daboval T, Dunn S. Quality appraisal of Canadian guidelines on the perinatal care of potential extremely premature infants: how do they rate? Paediatr Child Health (Oxford). 2014;19:e68-e9.

[60] Nagler EV, Vanmassenhove J, Van der Veer SN, Nistor I, Van Biesen W, Webster AC, et al. Diagnosis and treatment of hyponatraemia: a systematic review of clinical practice guidelines. Nephrol Dial Transplant. 2013;28:i386.

[61] Origasa H. Critical appraisal of the Japanese guideline for the management of stroke. Value Health. 2009;12:A345.

[62] Padjas A, Schünemann H, Bousquet J, Brozek J. Assessment of the quality of methodological rigour and reporting of clinical practice guidelines for the management of allergic rhinitisdqugar study. World Allergy Organ J. 2012;5:S97-S8.

[63] Parisi P. Quality of clinical guidelines in pediatric headache. Ital J Pediatr. 2014;40:A86.

[64] Pearson EJM, McKinstry CE, Morris ME. Which clinical practice guideline for cancer-related fatigue is the most suitable for application in Australia? Asia Pac J Clin Oncol. 2015;11:111.

[65] Piano V, Schalkwijk A, Burgers J, Varrassi G, Kress H, Verhagen C, et al. Assessment with agree II of European clinical practice guidelines for the treatment of neuropathic pain in cancer patients. Eur J Pain Suppl. 2011;5:268.

[66] Pregno S, Trevisiol C. How to choose recommendations from different guidelines the available methodological instruments. Tumour Biol. 2011;32:S46.

[67] Rodriguez-Martinez CE, Sossa-Briceno MP, Acuna-Cordero R. Quality assessment of acute viral bronchiolitis clinical practice guidelines. Eur Respir J. 2015;46:Abstract PA1334.

[68] Rubtsova I, Shilkina O, Lishchyshyna O. Towards creating a national system of care for patients with orphan diseases. Value Health. 2015;18:A676.

[69] Sanchez E, Rossi F. Concerns on who guidelines of treating and preventing HIV infection. Value Health. 2015;18:A247.

[70] Santos F, Sola I, Rigau D, Arevalo-Rodriguez I, Seron P, Alonso-Coello P, et al. Quality assessment of clinical practice guidelines for the prescription of antidepressant drugs during pregnancy. J Popul Ther Clin Pharmacol. 2011;18:e185-e6.

[71] Savoia H, Roberts J, Abeypala W, Challis D, Clarke M, Earnshaw L, et al. Evidence-based patient blood management guidelines for obstetric and maternity patients. BJOG. 2015;122:302.

[72] Schalkwijk A, Piano V, Hekster Y, Kress H, Lanteri-Minet M, Burgers J, et al. Comparative analysis of european recommendations and evidence analysis of treatment of neuropathic pain in cancer patients with the linker procedure. Palliat Med. 2012;26:483.

[73] Schieir O, Hazlewood G, Akhavan P, Bykerk V, Bombardier C. Adapting ADAPTE: a novel methodology for the development of national clinical practice guidelines. J Rheumatol. 2010;37:1315.

[74] Schildmann E, Schildmann J. Palliative sedation therapy: a limited systematic review on content and quality of guidelines. Palliat Med. 2010;24:S96.

[75] Schildmann EK, Schildmann JA, Dietz I. Palliative sedation therapy (PST) guidelines: findings from a systematic review and quality assessment. Palliat Med. 2014;28:566-7.

[76] Schmidt S, Manns MP, Greten TF. Qualitative analysis of international guidelines for diagnosis and treatment of hepatocellular carcinoma by the agree instrument (Appraisal of Guidelines for Research and Evaluation). J Hepatol. 2010;52:S233.

[77] Schoenmaker N, Tromp M, Van der Lee H, Offringa M, Groothoff J. Current guidelines for the management of chronic dialysis in children: a systematic review. Pediatr Nephrol. 2011;26:1594-5.

[78] Schrijvers D, Rosselli Del Turco M, Marotti L, Maddock C. European cancer guidelines: a survey. Eur J Cancer. 2011;47:S267.

[79] Shikata S, Sato T, Miyazaki K, Okumura A, Takagaki N, Nakayama T, et al. Description of palliative medicine in guidelines for digestive system cancer medical care. J Clin Oncol. 2010;28:Abstract e19628.

[80] Siddiqui J, Sherren PB, Birchall MA. Evidence-based guidelines and protocols for the management of adult patients with a tracheostomy: a systematic review. Crit Care. 2013;17:S62.

[81] Simmons CE, Hogeveen S, Nichols J, Trudeau-Tavara S, Quan M. Quality and consistency in breast cancer clinical guidelines internationally: are we globally consistent? J Clin Oncol. 2010;28:Abstract 6096.

[82] Skidmore B, Dupuis-Leon R, Chari R, Wilson RD, Blake J. When the need for change is evident: a review of Canadian Ob/Gyn clinical practice guidelines. Int J Gynaecol Obstet. 2015;131:E254.

[83] Spuls PHI. Critical appraisal of treatment guidelines for psoriasis. Br J Dermatol. 2012;167:e5-e6.

[84] Ste-Marie PA. Developing the 2012 fibromyalgia guidelines: lessons learned. Pain Res Manag. 2013;18:e15.

[85] Stergiou-Kita M, Dawson D, Rappolt S, Beaton D. Developing a guideline for vocational evaluation following traumatic brain injury: the systematic review. Arch Phys Med Rehabil. 2010;91:e7-e8.

[86] Stergiou-Kita M, Dawson D, Rappolt S, Hunt A. The inter-professional clinical practice guideline for vocational evaluation following traumatic brain injury: developing systematic and evidence-based approaches. Brain Inj. 2012;26:394-5.

[87] Stoffer M, Taurok D, Prodinger B, Smolen J, Woolf A, Stamm T. Are occupational therapy interventions included in the most commonly used European clinical-practice guidelines for the management of osteoarthritis? Ann Rheum Dis. 2012;71:754.

[88] Stone MA, Wilkinson JC, Charpentier G, Clochard N, Lindblad U, Muller UA, et al. Appraisal and comparison of guidelines for the management of people with type 2 diabetes in eight European countries. Diabetologia. 2009;52:S101.

[89] Szegesdi I, Kis E, Zimanyi M, Vimlati L, Kemeny L. Assessment of the scope and quality of clinical practice guidelines in burn injury. Eur J Anaesthesiol. 2009;26:16.

[90] Thompson GC, Ali S, Neto G, Gouin S, McClennan S, Pearce J, et al. Developing guidelines for the early recognition and emergency management of severe sepsis in children. Paediatr Child Health (Oxford). 2011;16:49A-50A.

[91] Tong A, Chapman J, Wong G, De Bruijn J, Craig JC. Screening and follow up of living kidney donors: a systematic review of clinical practice guidelines. Am J Transplant. 2012;12:507.

[92] Tremblay M, LeBlanc A, Carson V, Connor Gorber S, Duggan M, Janssen I, et al. Development and launch of the Canadian Physical Activity Guidelines for the Early Years. J Sci Med Sport. 2012;15:S325-S6.

[93] Tunnicliffe DJ, Kim S, Singh-Grewal D, Phoon RKS, Craig JC, Tong A. Diagnosis, monitoring and treatment of lupus nephritis: systematic review of clinical practice guidelines. Nephrology. 2014;19:38.

[94] Van Royen K, Remmen R, Vanmeerbeek M, Peremans L. Collaborative care in the management of addiction to alcohol, illegal drugs, hypnotics and tranquilizers: a review. Eur J Gen Pract. 2013;19:34.

[95] Vogel G, Eldh AC. Evidence as a basis for clinical practice guidelines on mechanical ventilation in intensive care in Sweden. Intensive Care Med. 2012;38:S225.

[96] Weinmann S. European and international guidelines on schizophrenia: consistencies and disagreements. Eur Arch Psychiatry Clin Neurosci. 2009;259:S20-S1.

[97] White P, Finch C. An assessment of the 2008 Zurich consensus statement on concussion in sport using the Appraisal of Guidelines for Research and Evaluation II (AGREE II). J Sci Med Sport. 2013;16:e22.

[98] Wiegerinck M, Huigen S, Van Barneveld T, Mol BW, Duvekot H. Comparing international guidelines on hypertensive disorders in pregnancy. Am J Obstet Gynecol. 2012;206:S283.

[99] Xiaofeng Kang X, Li Z, Himmelfarb CD, Zhang J, Li Q, Lv R, et al. Quality appraisal of international clinical practice guidelines for managing acute heart failure. Eur J Heart Fail. 2015;17:424.

[100] Yamada J, Lee G, Kyololo O, Shorkey A, Stevens B. Systematic review of acute procedural pain guidelines for infants and children. J Pain. 2014;15:S68.

Multiple publication

None

No German- or English-language publication

[1] Calvache JA, Guzman EL, Buitrago LMG, Torres CG, Torres M, Buitrago G, et al. Evidence-based clinical practice manual: postoperative complications management. Revista Colombiana de Anestesiologia. 2015;43:51-60.

[2] Chen H, Li GL, Xu WT, Xu B. Quality assessment of clinical practice guidelines of acupuncture in China. Chinese Journal of Evidence-Based Medicine. 2014;14:772-5.

[3] Chen Y, Hu SL, Li YP, Shen G, Yan BX, Wang L. Global guidelines concerning pharmacological intervention for complicated hypertension: a systematic review. Chinese Journal of Evidence-Based Medicine. 2012;12:1446-62.

[4] Chen Y, Hu SL, Li YP, Shen G, Yan BX, Wang L. Guidelines concerning pharmacological intervention in simple hypertension: a systematic review. Chinese Journal of Evidence-Based Medicine. 2012;12:1180-94.

[5] Cui SC, Hou HY, Li YP, Chen YQ, Lan XX. Clinical practice guidelines on infertility: a systematic review. Chinese Journal of Evidence-Based Medicine. 2013;13:947-54.

[6] Wei ML, Liu M. Analysis of clinical guidelines developed based on evidence in China. Chinese Journal of Evidence-Based Medicine. 2013;13:927-32.

[7] Zhang LL, Li YP, Zhang C, Huang L, Liang Y, Han L. Analysis on status of clinical guidelines and evaluation on evidence-based guidelines of children in China. Chinese Journal of Evidence-Based Medicine. 2011;11:991-9.
