# Supplementary material for: Guideline appraisal with AGREE II: Systematic review of the current evidence on how users handle the 2 overall assessments
Source: PLoS One. 2017 Mar 30;12(3):e0174831. doi: 10.1371/journal.pone.0174831 (PMC5373625; doi:10.1371/journal.pone.0174831)
Supplement: S4 File — (PDF) [file pone.0174831.s004.pdf]

S4: Characteristics of guidelines included in the publications

Author / Year	Aim of publication	Number of assessors	Number of guidelines appraised with AGREE II	Publication dates of guidelines included	Guideline topic
Abdelsattar 2015	▪ Assessment of the methodological quality of guidelines<br>▪ Assessment of the evidence base of guidelines<br>▪ Comparison of recommendations of several guidelines	4	5	2011-2014	Colon cancer
Acuna-Izcaray 2013	Assessment of the methodological quality of guidelines	4	18	2001, 2003, 2005-2009	Asthma
Agrawal 2012	▪ Assessment of the methodological quality of guidelines<br>▪ Comparison of recommendations of several guidelines	2	10	2008-2010	Invasive fungal disease (haematological-oncological setting)
Al-Ansary 2013	▪ Assessment of the methodological quality of guidelines<br>▪ Comparison of recommendations of several guidelines	4	11	2006-2011	Hypertension
Altman 2015	▪ Assessment of the methodological quality of guidelines<br>▪ Comparison of recommendations of several guidelines	10	2	2003, 2008, 2009, 2012-2014	Osteoarthritis
Alvarez-Vargas 2015	Assessment of the methodological quality of guidelines	3	4	2013, 2014	Hypertension
Arevalo-Rodriguez 2013	Assessment of the methodological quality of guidelines	4	15	2005-2011	Dementia
Armstrong 2016	▪ Assessment of the methodological quality of guidelines<br>▪ Comparison of recommendations of several guidelines	3	19	2004, 2006, 2007, 2009, 2010, 2012, 2013, 2015	Osteoporosis
Avin 2015	Development of Clinical Guidance	3-5	5	2003, 2011-2013,	Prevention of falls

Author / Year	Aim of publication	Number of assessors	Number of guidelines appraised with AGREE II	Publication dates of guidelines included	Guideline topic
	Statements (CGS) on the basis of guidelines			2015	
Barber 2015	▪ Assessment of the methodological quality of guidelines<br>▪ Assessment of the quality indicators included in the guidelines	2	10	2009-2012	Prevention of cardiovascular diseases in patients with rheumatoid arthritis
Bekkering 2014	Identification of the current state of knowledge on the basis of high-quality guidelines	2	9	2007-2011	Alcohol and drug abuse
Binepal 2015	Assessment of the methodological quality of guidelines	3	16	2001, 2002, 2004-2006, 2008, 2009, 2011, 2012, 2014	Care of prematurely born infants
Birken 2015	Assessment of the methodological quality of guidelines	2	16	No details provided	Care of cancer survivors
Bragge 2014	Assessment of the methodological quality of guidelines	4	11	2003, 2005-2008, 2011	▪ Attention training<br>▪ Externals aids as memory aids<br>▪ Brain injury<br>▪ Cognitive rehabilitation<br>▪ Drug treatment of neurological complications of traumatic brain injury<br>▪ Neurogenic limitation<br>▪ Traumatic brain injury
Brosseau 2014a	▪ Assessment of the methodological quality of guidelines	2	11	2002, 2004, 2006, 2007, 2009, 2011	Rheumatoid arthritis
Brosseau 2014b	▪ Assessment of the methodological quality of guidelines<br>▪ Comparison of recommendations of	2 (3. Assessor is used as a	12	2001, 2003-2005, 2007-2008, 2010-2013	Osteoarthritis

Author / Year	Aim of publication	Number of assessors	Number of guidelines appraised with AGREE II	Publication dates of guidelines included	Guideline topic
	several guidelines	substitute)			
Burda 2014	Assessment of the methodological quality of WHO guidelines	3	124	2008-2012	▪ Disability<br>▪ Blood sampling / donation<br>▪ Diabetes mellitus<br>▪ Diarrhoea and pneumonia<br>▪ Abuse of legal and illegal drugs<br>▪ Diet and physical activity<br>▪ Cancer<br>▪ Air quality in buildings<br>▪ Notifiable diseases (including diagnostics)<br>▪ Women's health, contraception, pregnancy, birth, care of newborns<br>▪ Infection control<br>▪ Non-communicable diseases (not further specified)<br>▪ Physiotherapy<br>▪ Mental and neurological illnesses as well as disorders related to substance abuse<br>▪ Radon concentration in buildings<br>▪ Rehabilitation<br>▪ Pain<br>▪ Safe surgery<br>▪ Injuries (not further specified)<br>▪ Access to healthcare professionals<br>▪ 9 guidelines provided no

Author / Year	Aim of publication	Number of assessors	Number of guidelines appraised with AGREE II	Publication dates of guidelines included	Guideline topic
					details
Burnett 2014	▪ Comparison of recommendations of several guidelines<br>▪ Assessment of the methodological quality of guidelines	3	20	2002, 2004, 2006-2012	▪ Acute lymphatic leukaemia<br>▪ Autoimmune hepatitis<br>▪ Inflammatory bowel and skin diseases<br>▪ Rheumatic diseases
Cassis 2015	Assessment of the methodological quality of guidelines	2	55	2001-2015	Inherited neurometabolic disorders
Castellani 2015	Assessment of the methodological quality of guidelines	2	14	2005-2007, 2009-2013	Bipolar disorders
Chen 2015	Assessment of the methodological quality of guidelines	2	17	2001, 2004, 2007-2011	Hypertension
Chua 2015	▪ Assessment of the methodological quality of guidelines<br>▪ Comparison of recommendations of several guidelines	4	10	2009-2013, 2015	Urinary tract infection
Colebatch-Bourn 2015	Improvement of the quality of rheumatology guidelines	k. A.	27	2000, 2003, 2005-2014	Rheumatology
Damiani 2014	▪ Examination of the applicability of guidelines to the target population<br>▪ Examination of the association between guideline quality and the applicability to the target population	2	22	2001, 2002, 2005-2008, 2010-2012	Dementia
Dersch 2015	Assessment of the methodological quality of guidelines	2	8	1999, 2004, 2006, 2007, 2010, 2012	Lyme borreliosis
Don-Wauchope 2012	▪ Evaluation of the applicability of the AGREE II instrument to guidelines of the National Academy of Clinical Biochemistry<br>▪ Assessment of the methodological quality of guidelines of the National	2	11	2003, 2006, 2007, 2009, 2010	Clinical biochemistry

**Author / Year**	**Aim of publication**	**Number of assessors**	**Number of guidelines appraised with AGREE II**	**Publication dates of guidelines included**	**Guideline topic**
	Academy of Clinical Biochemistry				
Falconi 2015	Assessment of the methodological quality of guidelines	k. A.	8	2004-2007, 2012-2014	Cystic neoplasm of the pancreas
Fisher 2014	Development and implementation of guidelines	11	1	No details provided	Urinary incontinence
Fouche 2014	Assessment of the methodological quality of guidelines	2	1	2013	Arrhythmia
Gamst-Jensen 2014	▪ Assessment of the methodological quality of guidelines<br>▪ Comparison of recommendations of several guidelines	2	4	▪ 2007,<br>▪ No details for 2 guidelines	Burns
Gandhi 2015	▪ Assessment of the methodological quality of guidelines<br>▪ Comparison of recommendations of several guidelines	2	17	2006, 2007, 2009-2014	Breast cancer
Gillon 2014	▪ Assessment of the methodological quality of guidelines<br>▪ Comparison of recommendations of several guidelines	2	13	2005, 2007, 2008-2013	Hypertension in pregnancy
Girardis 2016	Assessment of the methodological quality of guidelines	4	5	2006, 2008, 2010, 2013	Analgosedation
Goyet 2014	▪ Identification of impeding factors for interventions on knowledge transfer in areas with low income<br>▪ Development of implementation measures for knowledge transfer in areas with low income	3	2	2013	Pneumonia
Grimmer 2014	Development and testing of a short checklist for the assessment of the methodological quality of guidelines	3	6	2007, 2009, 2011, 2012, 2013	Traumatic brain injury

Author / Year	Aim of publication	Number of assessors	Number of guidelines appraised with AGREE II	Publication dates of guidelines included	Guideline topic
Gupta 2015	▪ Assessment of the methodological quality of guidelines<br>▪ Comparison of recommendations of several guidelines	4	13	No details provided	Prostate cancer
Gutarra-Vilchez 2014	Assessment of the methodological quality of guidelines	3	14	2006, 2008-2011	Assisted reproductive technology
Haddadi 2015	Assessment of the methodological quality of guidelines	4	7	2009, 2011-2013	Breast cancer
Haran 2014	Comparison of recommendations of several guidelines	2	5	2006, 2009, 2011, 2012	Postpartum care
Harris 2012	▪ Assessment of the methodological quality of guidelines<br>▪ Comparison of recommendations of several guidelines	2	19	2001, 2003, 2005, 2007-2011	Breast cancer
He 2015	Assessment of the methodological quality of guidelines	4	21	2001, 2005, 2008-2014	Pancreatic cancer
Heine 2015	Assessment of the methodological quality of guidelines	2	6	▪ 2005, 2010-2012<br>▪ No details for 1 guideline	Sensory hearing disorder
Henig 2013	Assessment of the methodological quality and reliability of guidelines	3	20	2004, 2005, 2007-2009, 2011, 2012	▪ Pneumonia<br>▪ Urinary tract infection
Holmer 2013	Assessment of the methodological quality of guidelines	2	24	2007-2012	Diabetes mellitus type 2
Holvoet 2015	Assessment of the methodological quality of guidelines	5	21	2003, 2004, 2009-2015	Gastrointestinal and liver diseases
Horner 2014	▪ Assessment of the methodological quality of guidelines<br>▪ Comparison of recommendations of several guidelines	2	26	2006, 2008-2014	Cone Beam CT

Author / Year	Aim of publication	Number of assessors	Number of guidelines appraised with AGREE II	Publication dates of guidelines included	Guideline topic
Huang 2015	Assessment of the methodological quality of guidelines	2	5	1996, 2011, 2013	Androgenetic alopecia
Huang 2013	▪ Assessment of the methodological quality of guidelines<br>▪ Comparison of recommendations of several guidelines	4	10	2000, 2007, 2009-2013	Thyroid nodules and cancer
Jiang 2015	Assessment of the methodological quality of guidelines	2	49	2001-2012	Head and neck tumours
Jokhan 2015	Comparison of the quality of local guidelines with the quality of national guidelines	2	2	2010, 2011	Fetal movements
Joosen 2015	▪ Assessment of the methodological quality of guidelines<br>▪ Comparison of recommendations of several guidelines	2	14	2003, 2005-2011	Mental illness
Kawala 2014	Assessment of the methodological quality of guidelines	2	4	2009, 2012, 2013	Acne
Kim 2014	Description of the procedure for the update of a Korean guideline	2	6	No details provided	Helicobacter pylori infections
Kirby 2015	Assessment of the methodological quality of guidelines	No details provided	7	▪ 2006-2009, 2011, 2014<br>▪ No details for 1 guideline	Actinic keratosis
Koh 2013	Evaluation of guidelines of AASLD	3	22	1998, 2000-2005, 2008-2011	Liver diseases
Kredo 2012	▪ Assessment of the methodological quality of guidelines of the Southern African development community<br>▪ Comparison of recommendations of the Southern African development community with reference guidelines	2	5	2005, 2006, 2010	▪ Diarrhoea<br>▪ HIV<br>▪ Hypertension<br>▪ Malaria<br>▪ Pre-eclampsia / eclampsia

Author / Year	Aim of publication	Number of assessors	Number of guidelines appraised with AGREE II	Publication dates of guidelines included	Guideline topic
Lambert 2015	▪ Production of a systematic review of guidelines<br>▪ Assessment of the methodological quality of guidelines<br>▪ Comparison of recommendations of several guidelines	2	19	2004-2006, 2009, 2011, 2012	Perioperative fasting
Langton 2011a	Assessment of the methodological quality of eviQ guidelines	3	2	No details provided	▪ Breast cancer<br>▪ Sarcoma
Langton 2011b	Assessment of the methodological quality of guidelines	3	21	No details provided	▪ Breast cancer<br>▪ Sarcoma
Larmer 2014	Assessment of the methodological quality of guidelines	4	17	2001, 2003, 2005, 2007-2009, 2011-2013	Osteoarthritis
Lee 2014	▪ Assessment of the methodological quality of guidelines<br>▪ Formulation of recommendations on the implementation of guidelines	2	18	2001, 2006, 2009-2013	Acute procedural pain
Li 2015	▪ Assessment of the methodological quality of guidelines<br>▪ Comparison of recommendations of several guidelines	k. A.	14	2003, 2005, 2007, 2009-2012	Pancreatic cancer
Li 2016	▪ Assessment of the methodological quality of guidelines<br>▪ Comparison of recommendations of several guidelines	4	8	2011-2014	Lung cancer
Loder 2012	▪ Assessment of the methodological quality of guidelines<br>▪ Comparison of recommendations of several guidelines	3	3	2009, 2012	Migraine
Lopez-Vargas 2013	▪ Assessment of the methodological quality of guidelines	2	11	2002, 2004, 2006, 2008, 2009, 2011	Renal disease

Author / Year	Aim of publication	Number of assessors	Number of guidelines appraised with AGREE II	Publication dates of guidelines included	Guideline topic
	▪ Comparison of recommendations of several guidelines				
Luitjes 2013	▪ Assessment of the methodological quality of guidelines<br>▪ Comparison of recommendations of several guidelines	55	6	2002, 2004, 2005, 2008, 2010	Hypertension in pregnancy
Lytras 2014	Assessment of the methodological quality of guidelines	6	7	1992, 1995, 1998, 2005, 2008, 2012	Asthma
Marciano 2014	▪ Evaluation of the evidence base of guidelines<br>▪ Assessment of the methodological quality of guidelines<br>▪ Comparison of recommendations of several guidelines	4	9	2003, 2006-2008, 2010-2012	Melanoma
Nagler 2014	Comparison of recommendations of several guidelines	4	10	2003-2004, 2007, 2008-2009, 2010-2014	Hyponatraemia
Nelson 2014	Assessment of the methodological quality of guidelines	2	16	2003, 2005, 2007-2009, 2012, 2013	Osteoarthritis
Norberg 2012	▪ Examination of the level of knowledge of guideline users concerning the guideline recommendations<br>▪ Survey of guidelines users' opinion on guideline quality	4	7	1999, 2006, 2008, 2009	Cannabis abuse
Nowobilski 2013	▪ Assessment of the methodological quality of guidelines<br>▪ Comparison of recommendations of guidelines	3	2	2009, 2011	Asthma
Nuckols 2014	▪ Assessment of the methodological quality of guidelines<br>▪ Comparison of recommendations of	4-6	13	2002, 2008-2012	Chronic pain

Author / Year	Aim of publication	Number of assessors	Number of guidelines appraised with AGREE II	Publication dates of guidelines included	Guideline topic
	guidelines				
Nuki 2014	▪ Assessment of the methodological quality of guidelines<br>▪ Comparison of recommendations of guidelines	2	4	2006, 2007, 2012, 2014	Gout
Olivera 2015	Assessment of the methodological quality of guidelines	2	5	2005, 2010-2012	Chagas disease
Padjas 2014	Assessment of the methodological quality of guidelines	4	10	2000, 2006-2008, 2010, 2011	Allergic rhinitis
Pak 2014	▪ Assessment of the methodological quality of guidelines<br>▪ Comparison of recommendations of several guidelines	2	3	2013	Hypertension
Parisi 2014	▪ Assessment of the methodological quality of the process of guideline development with AGREE II<br>▪ Identification of consistent and inconsistent recommendations	11	6	2002-2004, 2012	Headache in children
Piano 2013	Assessment of the methodological quality of guidelines	2	9	2006-2010	Neuropathic pain in cancer patients
Polus 2012	Assessment of the methodological quality of guidelines	4	4	2007, 2009, 2011	Maternal health care
Qaseem 2013	Assessment of the methodological quality of guidelines	4	4	2008-2010	Prostate cancer
Rapoport 2015	Assessment of the methodological quality of guidelines	8	9	2009-2013	Driving with an illness
Rios 2014	Assessment of the methodological quality of guidelines	3	10	2000, 2002, 2005, 2007, 2008, 2010, 2012	Bleeding of oesophageal or gastric varices
Rohde 2013	Assessment of the methodological	2	19	2003, 2005, 2006	Stroke

Author / Year	Aim of publication	Number of assessors	Number of guidelines appraised with AGREE II	Publication dates of guidelines included	Guideline topic
	quality of guidelines			,2008-2010	
Sabharwal 2014a	Assessment of the methodological quality of AAOS guidelines	3	14	2007, 2009-2013	Orthopaedics
Sabharwal 2014b	Assessment of the methodological quality of guidelines	2	7	2009-2013	Thrombosis prophylaxis for orthopaedic surgery
San Martin-Galindo 2015	Assessment of the methodological quality of guidelines	2	3	2004, 2005, 2012	Fissure sealing
Sanclemente 2014	Assessment of the methodological quality of guidelines	3	6	2007, 2009, 2011, 2012	Acne
Santos 2012	Assessment of the methodological quality of guidelines	4	12	2000, 2002, 2005-2010	▪ Anxiety disorders<br>▪ Bipolar affective disorders<br>▪ Depression<br>▪ Mental disorders (not further specified)
Schildmann 2015	▪ Comparison of recommendations of several guidelines<br>▪ Assessment of the methodological quality of guidelines	2	9	2003-2005. 2007, 2009, 2010, 2012	Palliative sedation
Schoenmaker 2013	▪ Assessment of the methodological quality of guidelines<br>▪ Comparison of recommendations of several guidelines	3	17	2000-2003, 2005-2007, 2009, 2011	Dialysis
Seron 2014	▪ Assessment of the methodological quality of guidelines<br>▪ Comparison of recommendations of several guidelines	3	9	1995, 1999, 2000, 2002, 2004, 2006, 2007	Cardiologic rehabilitation
Shen 2014	▪ Assessment of the methodological quality of guidelines	2	5	2003, 2008, 2009, 2011	Cushing syndrome

Author / Year	Aim of publication	Number of assessors	Number of guidelines appraised with AGREE II	Publication dates of guidelines included	Guideline topic
	▪ Comparison of guideline recommendations				
Simons 2016	▪ Comparison of recommendations of several guidelines<br>▪ Assessment of the methodological quality of guidelines	2	7	2011-2014	Chronic heart failure
Smith 2015	▪ Assessment of the methodological quality of guidelines<br>▪ Comparison of recommendations of several guidelines	4	3	2009, 2011-2013	Juvenile idiopathic arthritis
Stacey 2013	Development and evaluation of evidence-informed clinical nursing protocols for remote assessment, triage and support of cancer treatment-induced symptoms	4	12	2002, 2004, 2007, 2008	Cancer treatment-induced symptoms
Syan 2016	▪ Assessment of the methodological quality of guidelines<br>▪ Comparison of guideline recommendations	2	5	No details provided	Urinary incontinence
Tian 2015	Assessment of the methodological quality of guidelines	4	15	1997, 1998, 2003, 2007-2012	Glioblastoma
Tremblay 2010	▪ Description of the update process of the Canadian Physical Activity Guidelines	1	1	No details provided	Physical activity
Tudor 2013	Assessment of the methodological quality of guidelines	2	7	2005, 2006, 2008, 2010	Neurology
Tunnicliffe 2015	▪ Assessment of the methodological quality of guidelines<br>▪ Comparison of recommendations of guidelines	2	14	1999, 2008, 2010, 2012-2014	Lupus erythematosus
Vanclooster	▪ Assessment of the methodological	7	3	2007, 2010, 2011	Haemochromatosis

Author / Year	Aim of publication	Number of assessors	Number of guidelines appraised with AGREE II	Publication dates of guidelines included	Guideline topic
2015	quality of guidelines<br>▪ Comparison of recommendations of several guidelines				
Vanommeslaeghe 2015	▪ Assessment of the methodological quality of guidelines<br>▪ Comparison of recommendations of several guidelines	4	15	2006, 2009 2011-2013	No details provided
Wang 2014	Assessment of the methodological quality of guidelines	3	40	1999, 2001, 2003-2005, 2007-2013	Hepatocellular carcinoma or liver metastases
Werner 2016	Assessment of the methodological quality of guidelines of the European Dermatology Forum (EDF)	4	25	▪ 2009-2014<br>▪ No details for 7 guidelines	Dermatology
White 2014	Assessment of the methodological quality of a guideline	18	1	2008	Concussion during sports
Wilby 2015	Assessment of the methodological quality of guidelines	4	20	1997, 2001, 2004, 2006-2013	Infectious diseases in children
Wong 2015	▪ Assessment of the methodological quality of guidelines<br>▪ Comparison of recommendations of several guidelines	2	17	1995, 2001-2004, 2007-2013	Treatment of injuries due to traffic accidents
Wu 2015a	Assessment of the methodological quality of guidelines	4	2	2008, 2013	Macular degeneration
Wu 2015b	Assessment of the methodological quality of guidelines	4	3	2009, 2010	Open-angle glaucoma
Wu 2015c	Assessment of the methodological quality of guidelines	4	3	2012, 2013	Diabetic retinopathy
Wu 2015d	Assessment of the methodological quality of guidelines	4	3	2008, 2010, 2011	Cataract
Yaman 2015	Assessment of the methodological quality of NASS guidelines	4	6	2008-2013	Disorders of the spine

Author / Year	Aim of publication	Number of assessors	Number of guidelines appraised with AGREE II	Publication dates of guidelines included	Guideline topic
Yan 2013	Assessment of the methodological quality of guidelines	2	10	2002, 2003, 2007-2010	▪ Hypertension<br>▪ Pheochromocytoma
Ye 2014	▪ Assessment of the methodological quality of guidelines<br>▪ Comparison of recommendations of several guidelines	2	12	2008-2011, 2013, no details for 1 guideline	Monitoring of vancomycin therapy
Yuwen 2015	Assessment of the methodological quality of guidelines	3	5	1996, 2002, 2008, 2011	Stroke
Zeng 2014	▪ Assessment of the methodological quality of guidelines<br>▪ Comparison of recommendations of guidelines	4	13	2005, 2006, 2008, 2009, 2012, 2013	Upper respiratory tract infection in children
Zhang 2014	Assessment of the methodological quality of guidelines	2	17	1999-2001, 2006-2012	Myasthenia gravis
