# Supplementary material for: Guideline appraisal with AGREE II: Systematic review of the current evidence on how users handle the 2 overall assessments
Source: PLoS One. 2017 Mar 30;12(3):e0174831. doi: 10.1371/journal.pone.0174831 (PMC5373625; doi:10.1371/journal.pone.0174831)
Supplement: S5 File — (PDF) [file pone.0174831.s005.pdf]

S5: Information on the performance of the 2 overall assessments of AGREE II

**Author / Year**	**Assessment of overall guideline quality provided**	**Recommendation for use provided**	**Deviation from AGREE II (overall guideline quality)**	**Deviation from AGREE II (recommendation for use)**
Abdelsattar 2015	-	-	-	-
Acuna-Izcaray 2013	-	X	-	Specification of a fixed cut-off on the basis of standardized domain scores:<br>▪ “strongly recommended“ (standardized domain scores > 60%)
Agrawal 2012	X	X	no	no
Al-Ansary 2013	X	X	no	no
Altman 2015	-	-	-	-
Alvarez-Vargas 2015	X	X	no	no
Arevalo-Rodriguez 2013	-	-	-	-
Armstrong 2016	-	-	-	-
Avin 2015	-	X	-	no
Barber 2015	-	X	-	no
Bekkering 2014	-	-	-	-
Binepal 2015	X	-	no	-

Author / Year	Assessment of overall guideline quality provided	Recommendation for use provided	Deviation from AGREE II (overall guideline quality)	Deviation from AGREE II (recommendation for use)
Birken 2015	X	X	no	Specification of a fixed cut-off on the basis of standardized domain scores and overall guideline quality:<br>▪ "strongly recommended" (standardized score ≥ 50% in all domains)<br>▪ "recommended" (overall assessment ≥ 50%)<br>▪ "not recommended" (standardized score of < 50% in all domains; overall assessment < 50%)
Bragge 2014	X	-	no	-
Brosseau 2014a	X	X	no	Specification of a fixed cut-off on the basis of standardized domain scores:<br>▪ "recommended" (standardized domain scores > 60% in 3 out of 6 domains + domain 3 [rigour of development])
Brosseau 2014b	X	X	no	Specification of a fixed cut-off on the basis of standardized domain scores:<br>▪ "recommended" (standardized domain scores > 60% in domain 3 + 3-4 other domains)
Burda 2014	X	X	no	Consensus of individual assessments
Burnett 2014	X	-	Overall assessment calculated	-
Cassis 2015	X	X	-	Specification of a fixed cut-off on the basis of standardized domain scores and overall guideline quality:<br>▪ "strongly recommended" (standardized domain score ≥ 50% in all domains; standardized domain score ≥ 50% in 5 domains + standardized domain score 40% to 50% in 1 domain)

Author / Year	Assessment of overall guideline quality provided	Recommendation for use provided	Deviation from AGREE II (overall guideline quality)	Deviation from AGREE II (recommendation for use)
				▪ "recommended" (overall assessment ≥ 50%)<br>▪ "not recommended" (overall assessment < 50%)
Castellani 2015	-	X	no	no
Chen 2015	-	X	no	Specification of a fixed cut-off on the basis of standardized domain scores and overall guideline quality:<br>▪ "recommended" (standardized domain score ≥ 60% in all domains)<br>▪ "recommended with modifications" (standardized domain score ≥ 30% in 3 domains + standardized domain score < 60% in 1 domain)<br>▪ "not recommended" (standardized domain score < 30% in 3 domains)
Chua 2015	X	-	Overall assessment calculated	-
Colebatch-Bourn 2015	X	X	no	no
Damiani 2014	-	-	-	-
Dersch 2015	X	X	no	Specification of a fixed cut-off on the basis of standardized domain scores:<br>"not recommended" (standardized domain score < 50% in all domains)
Don-Wauchope 2012	X	X	no	no

Author / Year	Assessment of overall guideline quality provided	Recommendation for use provided	Deviation from AGREE II (overall guideline quality)	Deviation from AGREE II (recommendation for use)
Falconi 2015	X	X	no	no
Fisher 2014	-	X	-	no
Fouche 2014	-	-	-	-
Gamst-Jensen 2014	-	-	-	-
Gandhi 2015	X	X	no	no
Gillon 2014	X	X	no	no
Girardis 2016	X	-	no	no
Goyet 2014	X	-	no	-
Grimmer 2014	X	-	no	-
Gupta 2015	-	-	-	-
Gutarra-Vilchez 2014	-	X	-	Specification of a fixed cut-off on the basis of standardized domain scores:<br>▪ “recommended” (above 60%),<br>▪ “recommended with modifications” (30-60%),<br>▪ "not recommended" (below 30%).
Haddadi 2015	X	-	Overall assessment calculated	-
Haran 2014	X	-	Overall assessment calculated	-
Harris 2012	-	-	-	-
He 2015	-	-		
Heine 2015	X	X	no	no

Author / Year	Assessment of overall guideline quality provided	Recommen dation for use provided	Deviation from AGREE II (overall guideline quality)	Deviation from AGREE II (recommendation for use)
Henig 2013	-	-	-	-
Holmer 2013	-	X	-	Consensus of individual assessments
Holvoet 2015	-	-		
Horner 2014	-	-	-	-
Huang 2015	X	X	no	Specification of a fixed cut-off on the basis of standardized domain scores:<br>▪ "strongly recommended" (standardized domain scores > 60% in 4 domains)<br>▪ "recommended" (standardized domain scores from 30% to 60% in ≥ 4 domains + standardized domain scores ≥ 60% in 2 domains)<br>▪ "not recommended" (standardized domain scores < 30% in ≥ 4 domains)
Huang 2013	X	-	no	-
Jiang 2015	-	-	-	-
Jokhan 2015	X	-	no	-
Joosen 2015	X	-	no	no
Kawala 2014	-	-	-	-
Kim 2014	-	-	-	-
Kirby 2015	X	X	no	no
Koh 2013	X	-	no	-
Kredo 2012	-	-	-	-

Author / Year	Assessment of overall guideline quality provided	Recommendation for use provided	Deviation from AGREE II (overall guideline quality)	Deviation from AGREE II (recommendation for use)
Lambert 2015	X	-	no	-
Langton 2011a	X	-	no	-
Langton 2011b	X	-	no	-
Larmer 2014	-	X	-	no
Lee 2014	X	X	no	Specification of a fixed cut-off on the basis of overall guideline quality:<br>▪ “recommended” (overall score of 6 or 7)<br>▪ “recommended with modifications” (overall score of 3to 5)<br>▪ “not recommended” (overall score of 1 or 2).
Li 2015	-	X	-	Specification of a fixed cut-off on the basis of standardized domain scores:<br>▪ “strongly recommended” (standardized domain scores 60% in > 5 domains)<br>▪ “weakly recommended” (standardized domain scores 30% > 4 domains + standardized domain scores 60% in 1 domain)<br>▪ “not recommended” (standardized domain scores < 30% in > 3domains)
Li 2016	-	-	-	-
Loder 2012	X	X	no	no
Lopez-Vargas 2013	X	X	no	Specification of a fixed cut-off on the basis of overall guideline quality:<br>▪ “recommended” (overall score of 5 to 7)

**Author / Year**	**Assessment of overall guideline quality provided**	**Recommendation for use provided**	**Deviation from AGREE II (overall guideline quality)**	**Deviation from AGREE II (recommendation for use)**
				▪ “recommended with modifications” (overall score of 3 or 4)<br>▪ “not recommended” (overall score of 1 or 2).
Luitjes 2013	-	X	-	no
Lytras 2014	X	X	no	no
Marciano 2014	X	X	-	no
Nagler 2014	-	-	-	-
Nelson 2014	X	-	no	-
Norberg 2012	X	-	no	-
Nowobilski 2013	X	X	no	no
Nuckols 2014	X	X	no	no
Nuki 2014	X	-	no	-
Olivera 2015	-	-	-	-
Padjas 2014	-	-	-	-
Pak 2014	X	-	no	-
Parisi 2014	X	X	no	Specification of a fixed cut-off on the basis of standardized domain scores:<br>▪ “not recommended” (standardized domain score < 50%)
Piano 2013	-	-	-	-
Polus 2012	X	X	no	no

Author / Year	Assessment of overall guideline quality provided	Recommendation for use provided	Deviation from AGREE II (overall guideline quality)	Deviation from AGREE II (recommendation for use)
Qaseem 2013	X	X	no	no
Rapoport 2015	X	X	no	no
Rios 2014	X	X	Overall assessment calculated	Specification of a fixed cut-off on the basis of standardized domain scores:<br>▪ "recommended" (standardized domain score ≥ 70%)<br>▪ "not recommended" (standardized domain score < 70%)
Rohde 2013	X	X	no	Specification of a fixed cut-off on the basis of standardized domain scores:<br>▪ "recommended" (standardised domain score ≥ 66,67 %)<br>▪ "not recommended" (standardized domain score < 66,67 %)
Sabharwal 2014a	X	-	Overall assessment calculated	-
Sabharwal 2014b	X	X	no	no
San Martin-Galindo 2015	X	X	no	Specification of a fixed cut-off on the basis of standardized domain scores:<br>▪ "strongly recommended" (standardized domain scores > 60% in most domains)<br>▪ "recommended" (standardized domain scores from 30% to 60% in most domains)<br>▪ "not recommended" (standardized domain scores < 30% in most domains)
Sanclemente	-	X	-	Specification of a fixed cut-off on the basis of standardized

Author / Year	Assessment of overall guideline quality provided	Recommendation for use provided	Deviation from AGREE II (overall guideline quality)	Deviation from AGREE II (recommendation for use)
2014				domain scores:<br>▪ “recommended” (standardized domain scores ≥ 60 %)<br>▪ “not recommended” (standardized domain scores < 60 %)
Santos 2012	-	X	-	Specification of a fixed cut-off on the basis of standardized domain scores:<br>▪ “recommended” (standardized domain scores > 60%),<br>▪ “recommended with modifications” (standardized domain scores 30-60%),<br>▪ “not recommended” (standardized domain scores < 30%).
Schildmann 2015	-	-	-	-
Schoenmaker 2013	X	X	Overall assessment calculated	Specification of a fixed cut-off on the basis of standardized domain scores:<br>▪ “recommended” (standardized domain score ≥ 50%)<br>▪ “not recommended” (standardized domain score < 50%)
Seron 2014	-	X	-	Specification of a fixed cut-off on the basis of standardized domain scores with a general cut-off of 70% in all domains[a]
Shen 2014	-	X	-	Specification of a fixed cut-off on the basis of standardized domain scores:<br>▪ “recommended” (standardized domain scores > 60% in 4-6 domains),<br>▪ “recommended with modifications” (standardized domain scores 30-60% in most domains + standardized domain scores > 60% in 3 domains),

**Author / Year**	**Assessment of overall guideline quality provided**	**Recommendation for use provided**	**Deviation from AGREE II (overall guideline quality)**	**Deviation from AGREE II (recommendation for use)**
				▪ “not recommended” (standardized domain scores < 30% in most domains).
Simons 2016	X	-	no	-
Smith 2015	X	X	no	Specification of a fixed cut-off on the basis of standardized domain scores:<br>▪ “recommended” (standardized domain score 60 % in domain 3 + 2 other domains)
Stacey 2013	-	X	-	Reporting of the number of assessors recommending the guideline for use
Syan 2016	X	-	no	-
Tian 2015	-	-	-	-
Tremblay 2010	-	-	-	-
Tudor 2013	X	-	no	-
Tunnicliffe 2015	X	-	Overall assessment calculated	-
Vanclooster 2015	X	-	no	-
Vanommeslaeghe 2015	X	-	no	-
Wang 2014	-	X	-	Specification of a fixed cut-off on the basis of standardized domain scores:<br>▪ “strongly recommended” (standardized domain score > 60%

**Author / Year**	**Assessment of overall guideline quality provided**	**Recommendation for use provided**	**Deviation from AGREE II (overall guideline quality)**	**Deviation from AGREE II (recommendation for use)**
				in > 5 domains,<br>▪ “weakly recommended” (standardized domain scores > 30% in > 4 domains,<br>▪ “not recommended” (standardized domain scores < 30% in > 3 domains).
Werner 2016	X	X	no	no
White 2014	X	X	Overall assessment calculated	no
Wilby 2015	X	X	no	no
Wong 2015	-	-	-	-
Wu 2015a	X	-	no	-
Wu 2015b	X	-	no	-
Wu 2015c	X	-	no	-
Wu 2015d	X	-	no	-
Yaman 2015	X	-	Overall assessment calculated	-
Yan 2013	-	X	-	Specification of a fixed cut-off on the basis of standardized domain scores:<br>▪ “strongly recommended” (standardized domain score > 60% ≥4 domains),<br>▪ “recommended with modifications” (standardized domain scores 30-60% in most domains + standardized domain scores ≥ 60% in ≥ 2 domains),<br>▪ “not recommended” (standardized domain scores < 30% in most domains).

Author / Year	Assessment of overall guideline quality provided	Recommendation for use provided	Deviation from AGREE II (overall guideline quality)	Deviation from AGREE II (recommendation for use)
Ye 2014	-	X	-	no
Yuwen 2015	X	X	no	no
Zeng 2014	-	X	-	no
Zhang 2014	-	X		Specification of a fixed cut-off on the basis of standardized domain scores:<br>▪ "strongly recommended" (standardized domain score > 50% in 4 domains)<br>▪ "recommended" (standardized domain score > 50% in 3 domains)<br>▪ "weakly recommended" (standardized domain score > 50% in 1-2 domains)<br>▪ "not recommended" (standardized domain score < 50% in all domains)

x: Information provided in the publication.
-: No information provided in the publication.
