# Supplementary material for: Guideline appraisal with AGREE II: Systematic review of the current evidence on how users handle the 2 overall assessments
Source: PLoS One. 2017 Mar 30;12(3):e0174831. doi: 10.1371/journal.pone.0174831 (PMC5373625; doi:10.1371/journal.pone.0174831)
Supplement: S6 File — (PDF) [file pone.0174831.s006.pdf]

S6: Statistics for the multiple regression analysis

Analysis of variance

ANOVA[b]

Model		Sum of Squares	df	Mean Square	F	Sig.
1	Regression	216426.103	6	36071.017	327.181	.000[a]
	Residual	78496.575	712	110.248		
	Total	294922.678	718			

a. Predictors: (constant), Domain 6, Domain 4, Domain 5, Domain 2, Domain 1, Domain 3

b. Dependent variable: overall guideline quality

Collinearity statistics

Model		Tolerance	VIF
1	(Constant)		
	Domain 1	.516	1.937
	Domain 2	.494	2.023
	Domain 3	.378	2.647
	Domain 4	.570	1.756
	Domain 5	.670	1.492
	Domain 6	.644	1.552

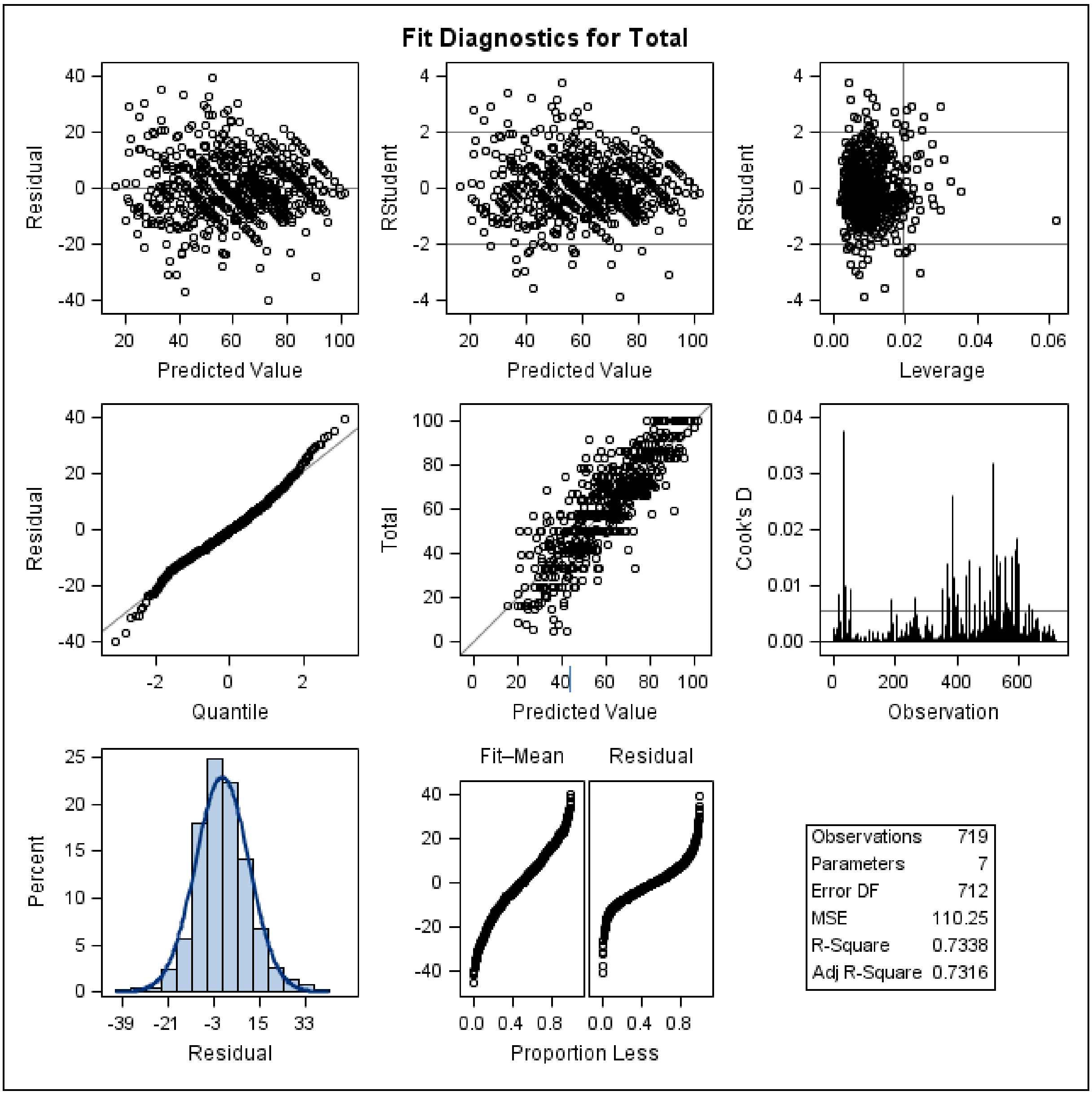
Fit Diagnostics for Total
Residual
Predicted Value
RStudent
Leverage
Quantile
Total
Cook's D
Observation
Percent
Fit–Mean
Proportion Less
Observations 719
Parameters 7
Error DF 712
MSE 110.25
R-Square 0.7338
Adj R-Square 0.7316

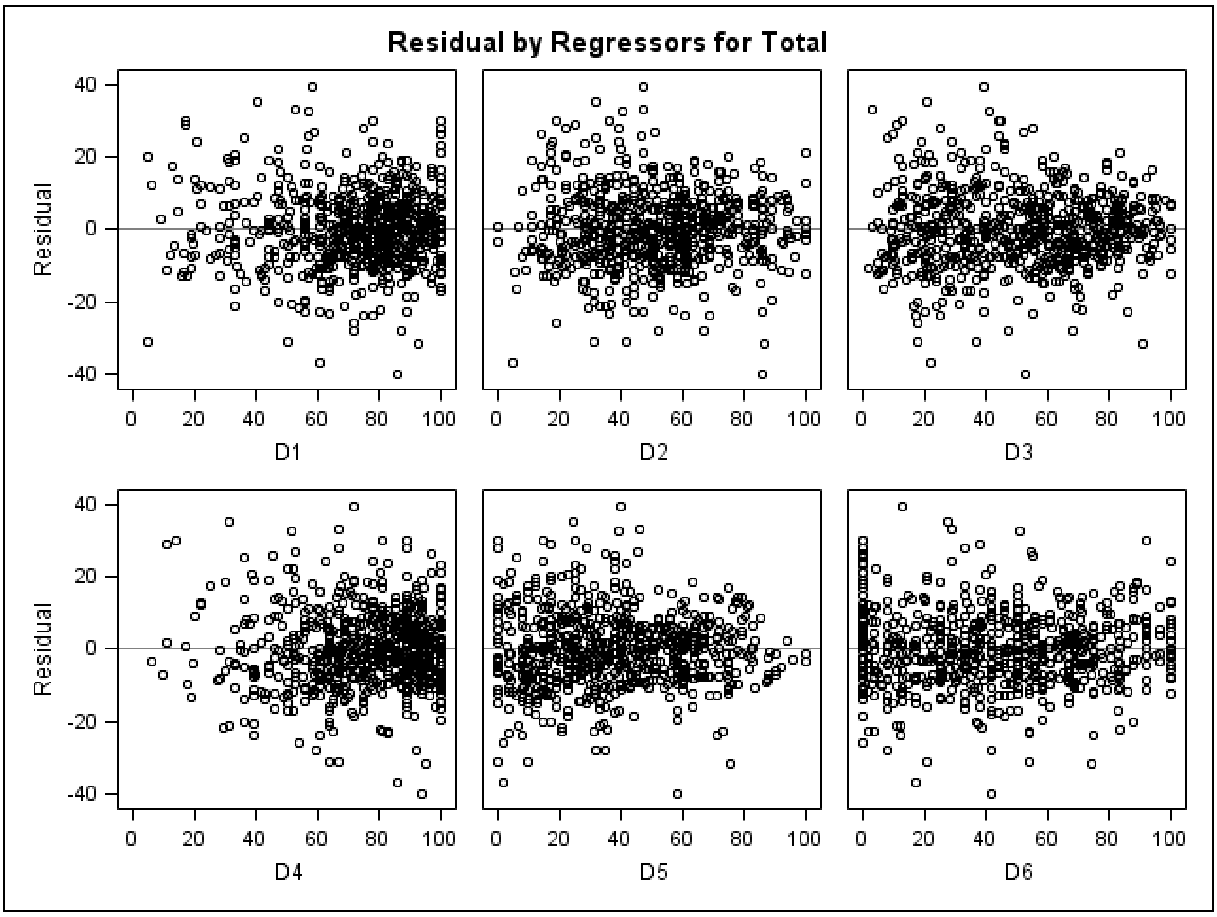

Residual by Regressors for Total
Residual
D1
D2
D3
D4
D5
D6
40
20
0
-20
-40
0
20
40
60
80
100

Correlations of independent variables

		Domain1	Domain 2	Domain 3	Domain 4	Domain 5	Domain 6
Pearson correlation	Domain 1	1.000	.555	.616	.573	.416	.342
	Domain 2	.555	1.000	.659	.467	.484	.446
	Domain 3	.616	.659	1.000	.561	.440	.571
	Domain 4	.573	.467	.561	1.000	.455	.315
	Domain 5	.416	.484	.440	.455	1.000	.385
	Domain 6	.342	.446	.571	.315	.385	1.000

Descriptive statistics

	Mean	Std. Deviation	N
Assessment of guideline quality	61.1585	20.26711	719
Domain 1	72.3956	21.10985	719
Domain 2	51.2753	21.24538	719
Domain 3	51.0951	25.04452	719
Domain 4	75.7952	19.42430	719
Domain 5	37.2841	23.19451	719
Domain 6	44.1308	28.72576	719

Model summary[b]

Model	R	R Square	Adjusted R Square	Std. Error of the Estimate
1	.857[a]	.734	.732	10.49990

a. Predictors: (constant), Domain 6, Domain 4, Domain 5, Domain 2, Domain 1, Domain 3

b. Dependent variable: Overall assessment 1

Model		Unstandardized Coefficients		Standardized Coefficients	t	Sig.	Correlations		
		B	Std. Error	Beta			Zero-order	Partial	Part
1	(Constant)	5.591	1.753		3.190	.001			
	Domain 1	.175	.026	.183	6.784	.000	.668	.246	.131
	Domain 2	.062	.026	.065	2.381	.018	.634	.089	.046
	Domain 3	.300	.025	.371	11.796	.000	.771	.404	.228
	Domain 4	.203	.027	.194	7.583	.000	.652	.273	.147
	Domain 5	.163	,021	.187	7.913	.000	.582	.284	.153
	Domain 6	.065	.017	.093	3.841	.000	.529	.142	.074

Residuals statistics[a]

	Minimum	Maximum	Mean	Std. Deviation	N
Predicted value	15,9166	101,5885	61,1585	17,36171	719
Residual	-40,21762	39,29159	,00000	10,45594	719
Std. predicted value	-2,606	2,329	,000	1,000	719
Std. residual	-3,830	3,742	,000	,996	719

a. Dependent variable: overall guideline quality
