# Supplementary material for: Guideline appraisal with AGREE II: Systematic review of the current evidence on how users handle the 2 overall assessments
Source: PLoS One. 2017 Mar 30;12(3):e0174831. doi: 10.1371/journal.pone.0174831 (PMC5373625; doi:10.1371/journal.pone.0174831)
Supplement: S7 File — (PDF) [file pone.0174831.s007.pdf]

S7: Assessment of model quality of the multinomial regression analysis

Statistics for model adaption		
**Criterion**	**Constants only**	**Constants and covariates**
**AIC**	1561.621	1048.277
**SC**	1570.891	1113.162
**-2 LOG L**	1557.621	1020.277

**R square**	0.5064	**Max- newly scaled R square**	0.5815

Test – global null hypothesis: BETA=0			
**Test**	**Chi-square**	**DF**	**Pr > ChiSq**
**Likelihood ratio**	537.3440	12	<.0001
**Score**	406.5291	12	<.0001
**Wald**	247.0930	12	<.0001

Pearson correlation coefficients, N = 761						
**Domain**	**D1**	**D2**	**D3**	**D4**	**D5**	**D6**
**D1**	1.000	0.554	0.589	0.544	0.371	0.416
**D2**2	0.554	1.000	0.670	0.492	0.534	0.435
**D3**3	0.589	0.670	1.000	0.571	0.484	0.564
**D4**4	0.544	0.492	0.571	1.000	0.448	0.370
**D5**5	0.371	0.534	0.484	0.448	1.000	0.427
**D6**	0.416	0.435	0.564	0.370	0.427	1.000
