# Supplementary material for: Guideline appraisal with AGREE II: Systematic review of the current evidence on how users handle the 2 overall assessments
Source: PLoS One. 2017 Mar 30;12(3):e0174831. doi: 10.1371/journal.pone.0174831 (PMC5373625; doi:10.1371/journal.pone.0174831)
Supplement: S8 File — (PDF) [file pone.0174831.s008.pdf]

# PRISMA 2009 Checklist

| Section/topic | # | Checklist item | See section |
|---|---|---|---|
| **TITLE** | | | |
| Title | 1 | Identify the report as a systematic review, meta-analysis, or both. | Title (page one) |
| **ABSTRACT** | | | |
| Structured summary | 2 | Provide a structured summary including, as applicable: background; objectives; data sources; study eligibility criteria, participants, and interventions; study appraisal and synthesis methods; results; limitations; conclusions and implications of key findings; systematic review registration number. | Abstract (page 2 to 3) |
| **INTRODUCTION** | | | |
| Rationale | 3 | Describe the rationale for the review in the context of what is already known. | Introduction (page 4 to 6) |
| Objectives | 4 | Provide an explicit statement of questions being addressed with reference to participants, interventions, comparisons, outcomes, and study design (PICOS). | Introduction (page 6) |
| **METHODS** | | | |
| Protocol and registration | 5 | Indicate if a review protocol exists, if and where it can be accessed (e.g., Web address), and, if available, provide registration information including registration number. | It doesn't exist an explicit review protocol. All materials and methods are described in section "Materials and Methods" on pages 6 and 7. |
| Eligibility criteria | 6 | Specify study characteristics (e.g., PICOS, length of follow-up) and report characteristics (e.g., years considered, language, publication status) used as criteria for eligibility, giving rationale. | Materials and Methods (page 6) |
| Information sources | 7 | Describe all information sources (e.g., databases with dates of coverage, contact with study authors to identify additional studies) in the search and date last searched. | Materials and Methods (page 6) |
| Search | 8 | Present full electronic search strategy for at least one database, including any limits used, such that it could be repeated. | Materials and Methods (page 6) and Supporting information S1 |
| Study selection | 9 | State the process for selecting studies (i.e., screening, eligibility, included in systematic review, and, if applicable, included in the meta-analysis). | Materials and Methods (page 6) |
| Data collection process | 10 | Describe method of data extraction from reports (e.g., piloted forms, independently, in duplicate) and any processes for obtaining and confirming data from investigators. | Materials and Methods Section "Data extraction and analysis" (page 7) |
| Data items | 11 | List and define all variables for which data were sought (e.g., PICOS, funding sources) and any assumptions and simplifications made. | Materials and Methods Section "Data extraction and analysis" (page 7) |

# PRISMA 2009 Checklist

| Section/topic | # | Checklist item | See section |
|---|---|---|---|
| Risk of bias in individual studies | 12 | Describe methods used for assessing risk of bias of individual studies (including specification of whether this was done at the study or outcome level), and how this information is to be used in any data synthesis. | Since the extracted methods were presented exclusively with descriptive statistics and no quantitative synthesis of the data (such as Meta-Analyse) took place, no risk of bias assessment was conducted for the publications included. |
| Summary measures | 13 | State the principal summary measures (e.g., risk ratio, difference in means). | Materials and Methods (page 7) |
| Synthesis of results | 14 | Describe the methods of handling data and combining results of studies, if done, including measures of consistency (e.g., $I^2$) for each meta-analysis. | Materials and Methods (page 7)<br>No meta-analysis was carried out. |
| Risk of bias across studies | 15 | Specify any assessment of risk of bias that may affect the cumulative evidence (e.g., publication bias, selective reporting within studies). | Since the extracted methods were presented exclusively with descriptive statistics and no quantitative synthesis of the data (such as Meta-Analyse) took place, no risk of bias assessment was conducted for the publications included. |
| Additional analyses | 16 | Describe methods of additional analyses (e.g., sensitivity or subgroup analyses, meta-regression), if done, indicating which were pre-specified. | No additional analyses were carried out. |
| **RESULTS** | | | |
| Study selection | 17 | Give numbers of studies screened, assessed for eligibility, and included in the review, with reasons for exclusions at each stage, ideally with a flow diagram. | Results (page 7), Figure 1, Supporting Information S2 and S3 |
| Study characteristics | 18 | For each study, present characteristics for which data were extracted (e.g., study size, PICOS, follow-up period) and provide the citations. | Results (page 7), Figure 2, Supporting information S4 and S5 |
| Risk of bias within studies | 19 | Present data on risk of bias of each study and, if available, any outcome level assessment (see item 12). | Since the extracted methods were presented exclusively with descriptive statistics and no quantitative synthesis of the data (such as Meta-Analyse) took place, no risk of bias assessment was conducted for the publications included. |
| Results of individual studies | 20 | For all outcomes considered (benefits or harms), present, for each study: (a) simple summary data for each intervention group (b) effect estimates and confidence intervals, ideally with a forest plot. | Results (page 8), Figure 2, Supporting information S4 to S7<br>Since the extracted methods were presented exclusively with descriptive statistics and no quantitative synthesis of the data (such as Meta-Analyse) took place. The publications included are publications on applied methods (AGREE II) |

# PRISMA 2009 Checklist

| Section/topic | # | Checklist item | See section |
|---|---|---|---|
| Synthesis of results | 21 | Present results of each meta-analysis done, including confidence intervals and measures of consistency. | Materials and Methods (page 8 to 12).<br>No meta-analysis was carried out. |
| Risk of bias across studies | 22 | Present results of any assessment of risk of bias across studies (see Item 15). | Since the extracted methods were presented exclusively with descriptive statistics and no quantitative synthesis of the data (such as Meta-Analyse) took place, no risk of bias assessment was conducted for the publications included. |
| Additional analysis | 23 | Give results of additional analyses, if done (e.g., sensitivity or subgroup analyses, meta-regression [see Item 16]). | No additional analyses were carried out (see Item 16). |
| **DISCUSSION** | | | |
| Summary of evidence | 24 | Summarize the main findings including the strength of evidence for each main outcome; consider their relevance to key groups (e.g., healthcare providers, users, and policy makers). | Discussion (page 12 to 14) |
| Limitations | 25 | Discuss limitations at study and outcome level (e.g., risk of bias), and at review-level (e.g., incomplete retrieval of identified research, reporting bias). | Discussion section "strength and limitations" (page 14 to 16) |
| Conclusions | 26 | Provide a general interpretation of the results in the context of other evidence, and implications for future research. | Conclusions (page 16) |
| **FUNDING** | | | |
| Funding | 27 | Describe sources of funding for the systematic review and other support (e.g., supply of data); role of funders for the systematic review. | A funding statement is disclosed during the submission process. |

*From:* Moher D, Liberati A, Tetzlaff J, Altman DG, The PRISMA Group (2009). Preferred Reporting Items for Systematic Reviews and Meta-Analyses: The PRISMA Statement. PLoS Med 6(6): e1000097. doi:10.1371/journal.pmed1000097

For more information, visit: **www.prisma-statement.org**.
